# RIP140 regulates transcription factor HES1 oscillatory expression and mitogenic activity in colon cancer cells

**DOI:** 10.1002/1878-0261.13626

**Published:** 2024-03-08

**Authors:** Nour Sfeir, Marilyn Kajdan, Stéphan Jalaguier, Sandrine Bonnet, Catherine Teyssier, Samuel Pyrdziak, Rong Yuan, Emilie Bousquet, Antonio Maraver, Florence Bernex, Nelly Pirot, Florence Boissière‐Michot, Audrey Castet‐Nicolas, Marion Lapierre, Vincent Cavaillès

**Affiliations:** ^1^ IRCM, Institut de Recherche en Cancérologie de Montpellier France; ^2^ INSERM, U1194 France; ^3^ Université de Montpellier France; ^4^ Institut régional du Cancer de Montpellier France; ^5^ Department of Medical Microbiology, Immunology and Cell Biology, School of Medicine Southern Illinois University Springfield IL USA; ^6^ Translational Research Unit Montpellier Cancer Institute Val d'Aurelle France

**Keywords:** colorectal cancer, HES1, Notch pathway, RIP140

## Abstract

The transcription factor receptor‐interacting protein 140 (RIP140) regulates intestinal homeostasis and tumorigenesis through Wnt signaling. In this study, we investigated its effect on the Notch/HES1 signaling pathway. In colorectal cancer (CRC) cell lines, RIP140 positively regulated *HES1* gene expression at the transcriptional level via a recombining binding protein suppressor of hairless (RBPJ)/neurogenic locus notch homolog protein 1 (NICD)‐mediated mechanism. In support of these *in vitro* data, RIP140 and HES1 expression significantly correlated in mouse intestine and in a cohort of CRC samples, thus supporting the positive regulation of *HES1* gene expression by RIP140. Interestingly, when the Notch pathway is fully activated, RIP140 exerted a strong inhibition of *HES1* gene transcription controlled by the level of HES1 itself. Moreover, RIP140 directly interacts with HES1 and reversed its mitogenic activity in human CRC cells. In line with this observation, HES1 levels were associated with a better patient survival only when tumors expressed high levels of RIP140. Our data identify RIP140 as a key regulator of the Notch/HES1 signaling pathway, with a dual effect on *HES1* gene expression at the transcriptional level and a strong impact on colon cancer cell proliferation.

## Graphical abstract

The authors show that the transcription factor RIP140 exerts a dual effect on *HES1* gene transcription: an increase via an RBPJ/NICD‐mediated mechanism, and a strong inhibition controlled by the level of HES1 itself. Consequently, RIP140 participates in *HES1* oscillatory expression, and controls its mitogenic activity and prognostic value in breast cancers.

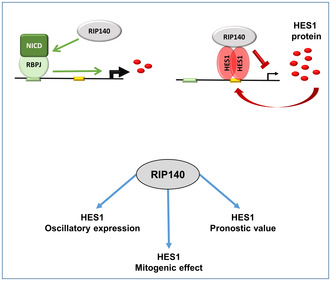

**Abbreviations**
APC: adenomatous polyposis coli
ChIP: chromatin immunoprecipitation
CRC: colorectal cancer
GST: glutathione‐S transferase
HES1: hes family bHLH transcription factor 1
NICD: Notch intracellular domain
NRIP1: nuclear receptor‐interacting protein 1
RBPJ: recombination signal binding protein for immunoglobulin kappa J region
RIP140: receptor‐interacting protein of 140 kDa
SOX9: SRY‐box transcription factor 9

## 1 Introduction

Colorectal cancer (CRC) is the third leading cause of cancer deaths with a mortality rate of 33% in developed countries [1]. Different genetic variations are involved in the development and progression of CRC [2]. In addition, various signaling pathways which regulate intestinal development and differentiation play important roles in early stages of colorectal carcinogenesis [3, 4].

Increasing evidence has shown that the Notch signaling pathway controls many aspects of intestinal epithelium development and renewal [5, 6]. The Notch pathway is an intercellular communication mechanism activated following the binding of a ligand of the jagged and delta family to a membrane receptor of the Notch family carried by the neighboring cell [7]. The interaction between a Notch receptor and its ligand leads to a conformational change of the receptor at the origin of a double sequential cleavage. The intracellular domain of the Notch receptor (NICD for Notch intracellular domain) is translocated to the nucleus to induce the expression of target genes (such as HES 1‐7 and HEY1‐2) with the aid of the transcription factor RBPJ and coactivators such as MAML1 (Mastermind‐like). In absence of Notch activation, RBPJ acts as a transcriptional repressor by recruiting a complex of co‐repressors which comprises SHARP (SMRT and HDAC associated repressor protein), CtBP (C‐terminal binding protein) and CtIP (CtBP interacting protein) [8].

The Notch signaling pathway plays an essential role in intestinal development and homeostasis by maintaining intestinal progenitors and stem cells in a state of proliferation while promoting their differentiation into absorptive cell lineage in the detriment of a secretory lineage differentiation [9]. In addition, the Notch pathway has an oncogenic potential in colon cancer [10]. One of its target genes, HES1, is the major effector in the intestine and plays an important role in CRC by regulating key parameters involved in tumorigenesis, including cell proliferation and differentiation [11, 12]. Interestingly, in order to avoid an aberrant activity of the Notch pathway, HES1 exerts a negative feedback loop on its own promoter leading to an autonomous oscillatory expression [13]. Finally, the promoter of the *HES1* gene is not only regulated by the Notch pathway but also by the Wnt pathway due to the presence of two TCF binding sites near the RBPJ site [14].

RIP140 (receptor‐interacting protein of 140 kDa) also known as NRIP1 (nuclear receptor‐interacting protein 1), was initially characterized as a transcriptional repressor of nuclear hormone receptors [15, 16]. We and other then identified RIP140 as a coregulator of various transcription factors, including E2F1 [17], HIFs [18], and NF‐KB [19]. The repressive activity of RIP140 involves several inhibitory domains interacting with histone deacetylases and CtBPs [20] and is controlled by different post‐translational modifications [21]. Using a mouse model lacking the *Rip140* gene, a wide range of physiological processes were shown to be regulated by RIP140, including female fertility [22] and mammary gland morphogenesis [23], fat metabolism [24], proinflammatory cytokine response [25] and cognition [26].

In the intestinal epithelium, our laboratory demonstrated that RIP140 inhibited the Wnt/β‐catenin signaling pathway and exerted an antiproliferative activity on CRC cells [27]. Consequently, RIP140 expression decreased in CRC samples as compared to the adjacent healthy tissue. Interestingly, in sporadic CRC, RIP140 mRNA and protein levels significantly correlated with a better overall survival of patients and was identified as a good prognosis marker [27, 28, 29]. More recently, we demonstrated that RIP140 acts as a major regulator of SOX9 signaling with functional relevance in intestinal physiopathology, in relation with Paneth cell differentiation and colon cancer cell proliferation [30]. In another recent study, we reported that RIP140 is involved in the regulation of microsatellite instability in CRC cells through the regulation of *MSH2* and *MSH6* gene expression [31]. Interestingly, a frame shift mutation in the RIP140 coding sequence was identified in microsatellite instable CRCs with a familial history and this mutation appeared to be associated with intestinal tumorigenesis.

In the present work, we demonstrated that RIP140 strongly interferes with the Notch pathway by controlling *HES1* gene expression at the transcriptional level, participating in the HES1 regulatory negative feedback loop, antagonizing HES1 mitogenic activity *in vitro* and impacting the prognostic value of HES1 in human CRC. Altogether, this work identifies RIP140 as a new regulator of the Notch/HES1 axis, which amplifies its role in the fine‐tuning of intestinal tumorigenesis.

## 2 Materials and methods

### 2.1 Cell culture

The human colon adenocarcinoma cell lines HT29 (RRID: CVCL_0320) and SW620 (RRID: CVCL_0547) were grown in DMEM‐F12 and RPMI medium, respectively. All media were supplemented with 10% FCS, 100 U·mL^−1^ penicillin, 100 mg·mL^−1^ streptomycin and 100 mg·mL^−1^ sodium pyruvate. The human colon cancer cell lines used in this study were obtained from the SIRIC Montpellier. Cell lines were routinely tested for mycoplasma contamination using the MycoAlert Plus assay (Lonza, Walkersville, MD, USA, Cat# LT07‐218). Short tandem repeat (STR) profiling was utilized to authenticate all the cell lines.

### 2.2 Plasmids and treatments

pRL‐TK‐renilla and pGL3 plasmids were obtained from Promega (Charbonnieres, France). Control vectors (pEF‐cmyc or pEGFP) pEF‐cmyc‐RIP140 and pEGFP‐RIP140 have been previously described [27]. The reporter plasmids containing the firefly luciferase gene under the control of different fragments of the *RIP140* promoter were already described [32]. The PCMV6‐HES1 [33], PCMV5‐NICD [34] and pRBPJ‐Luc plasmids were kindly given by the corresponding laboratories. The lentiviral expression vectors EF.hHES1.Ubc.GFP (LV‐HES1; #17624 [35]), EF.deltaBHES1.Ubc.GFP (DBD‐HES1 DNA‐binding domain mutant of HES1; #24982) were obtained from Addgene (Cambridge, MA, USA). The reporter plasmids containing the firefly luciferase gene under the control of various fragments of the *Hes1* promoter (2.5 kb; #43806 [36]), (467 bp; #41723 [37] or 467 bp lacking the RBPJ site; #43805) or the *Hes5* promoter (#41724 [37]) were obtained from Addgene. Different fragments of the Hes1 promoter (WT or mutRBPJ) were PCR amplified and cloned into pGL3‐basic plasmid previously digested with HindIII‐XhoI or HindIII‐KpnI to create I3, I1 and I1mutRBPJ reporter vectors which contained the −490/+46 or −125/+46 region of the RIP140 promoter with the wild‐type sequence or with a mutation of the RBPJ site. All the engineered PCR constructs were sequenced. Sp1‐Luc reporter and plasmids containing p21^WAF1/CIP1^ promoter (pWP101wild type) driving the luciferase reporter gene were provided by C. Seiser (Vienna BioCenter, Vienna, Austria) and Y. Sowa (Kyoto, Japan), respectively, and as previously described [38].

### 2.3 Luciferase and ChIP assays

Transient transfections with various promoter constructs were performed using Jet‐PEI® (275 ng of total DNA) according to the manufacturer instructions. SW620 and HT29 cells were seeded in 96‐well plates (3 × 10^4^ and 1.5 × 10^4^ cells per well respectively) 24 h prior to DNA transfection. Cells, in triplicates, were transfected with 25 ng of firefly luciferase‐based reporter constructs and 50 ng renilla luciferase plasmid pRL. In cotransfection experiments, different amounts of expression plasmids were added. The pRL‐TK‐Renilla plasmid (Ozyme®) was used to normalize the transfection efficiency. Firefly luciferase values were measured and normalized by the renilla luciferase activity. Values were expressed as relative luciferase activity (RLU) as mean ± SD.

ChIP assays at the *HES1* promoter were performed in HT29 cells using the CHIP‐IT kit (Active Motif, Carlsbad, CA, USA). Sonicated chromatin was immunoprecipitated with antibodies against IgG (sc‐3739, Santa Cruz Biotechnology, Inc, Heidelberg, Germany), H3pan (CC16310135, Diagenode, Liège, Belgium), and RIP140 (ab42126, Abcam, Paris, France). Immunoprecipitated DNA was amplified by qPCR using the primers listed in Table S1.

### 2.4 Small interfering RNA transfection

SW620 and HT29 cells were seeded in 6‐well plates (8 × 10^5^ and 5 × 10^5^ cells per well respectively) 24 h prior transfection. Small interfering RNA (siRNA) transfections targeting non‐relevant sequence (Ctl), RIP140 (100 pmol) or HES1 (200 pmol) were performed using INTERFERin^®^ transfection reagent. Cells were then incubated for 24 h before RNA analysis by quantitative RT‐qPCR.

### 2.5 Real‐time quantitative PCR (RT‐qPCR)

Total RNA was extracted from cells using Quick‐RNA kit (Zymo Research, Irvine, CA, USA) according to the manufacturer's instructions. RNA extraction from tissues or intestinal epithelial fraction was perform using EZNA^®^ Total RNA kit (Omega Bio‐tek) with bead column for more efficiency cell lyses. As previously described [27], total RNA (1 μg) was subjected to reverse‐transcription using qScript cDNA SuperMix (QuantaBio). RT‐qPCR were performed with the Roche LightCycler^®^ 480 instrument and the PerfeCTa SYBR Green FastMix (QuantaBio) and were carried out in a final volume of 10 μL using 0.25 μL of each primer (25 μm), 5 μL of the supplied enzyme mix, 2.5 μL of H_2_O and 2 μL of the template diluted at 1 : 6 (See Table S1 for primer sequences). After pre‐incubation at 95 °C, runs corresponded to 35 cycles of 15 s each at 95 °C, 5 s at 60 °C and 15 s at 72 °C. Melting curves of the PCR products were analyzed using the LightCycler^®^ software to exclude amplification of unspecific products. Results were normalized to RS9 or 28S housekeeping gene transcripts.

### 2.6 Immunoblotting

RIPA solution was used to extract cell proteins as previously described [39]. Cell extracts were analyzed after the migration of the 50 μg protein extract by Western blotting using a primary antibody against HES1 (#11988, Cell Signaling, Danvers, MA, USA). Signals were revealed using a rabbit peroxidase‐conjugated secondary antibody (1/5000, A6154 Sigma‐Aldrich^®^, St Louis, MO, USA) and a chemiluminescence substrate (ECL‐RevelBlotPlus; GE Healthcare^®^, Freiburg, Germany) according to the manufacturer's instructions. Protein quantifications were normalized with the β‐actin signal (A3854; Sigma‐Aldrich^®^).

### 2.7 DuoLink proximity ligation assay

The proximity ligation assay was performed to visualize interactions using the Duolink^®^ kit (Sigma‐Aldrich^®^) according to the manufacturer's instructions. As previously described [29], SW620 or HT29 cells were plated on slides (5 × 10^4^ cells per well) 24 h prior fixation with paraformaldehyde 3.7% and permeabilization with Triton X‐100 1%. After blocking with BSA 1% for at least 3 h, cells were incubated with two primary antibodies RIP140 (sc‐9459, Santa Cruz, Dallas, TX, USA) and HES1 (#11988, Cell Signaling Technology) overnight at 4 °C. A pair of oligonucleotide‐labeled secondary rabbit and goat antibodies IgG (Duolink^®^ In Situ PLA^®^ Probes) was used according to the manufacturer's instructions to bind to the primary antibodies. This pair of secondary antibodies generates a signal only when the two probes are in close proximity (40 nm). The PLA signals were assigned using the Duolink^®^ In Situ Detection Reagents Orange (554 nm laser line). Detection of the nucleus was done with the Hoechst (1/1000, Sigma‐Aldrich^®^). Slides were counterstained with Hoechst (1/1000, Sigma Aldrich^®^) and mounted with Mowiol (Sigma‐Aldrich^®^) for fluorescence microscopy. The images were obtained with ×40 magnification using an Axio Imager microscope (Carl‐Zeiss, Oberkochen, Germany).

### 2.8 GST pull‐down

Different fragments of RIP140 protein fused with GST: GST‐RIP1(27–439), GST‐RIP5 (428–739), GST‐RIP2 (683–1158) and the GST (Control) were produced and purified as previously described [20]. Unlabeled proteins were cell‐free‐synthesized using the TNT T7 Quick Reaction system according to the manufacturer's instructions (Promega) and incubated with purified GST fusion proteins coupled with glutathione sepharose 4B fast flow beads (GE Healthcare) in NETN buffer 20 mm Tris PH8, 100 mm NaCl, 1 mm EDTA, 0.5% Nonidet‐P40, 1 mm DTT, EDTA‐Free protease inhibitors (Complete^®^) overnight at 4 °C. After washing 4 times with NETN buffer, loading buffer 1× (50 μL) was added to the beads and boiled for 5 min. Proteins were separated on a 10% SDS/PAGE.

### 2.9 Animals

To generate the C57BL/6J mice line with conditional KO of the *Rip140* gene in the intestinal epithelium, RIPcKO^Int^ transgenic mice (RY‐Nrip1 strain obtained from the Yuan's lab, Springfield, IL, USA) were crossed with a mouse line expressing the CRE recombinase under the control of the villin promoter (vil‐Cre) [40] and with the tumor‐prone *Apc* ^flox^ mouse strain (C57BL/6‐Apctm1Tyj/J, Jackson Laboratory), obtained from the Fisher's lab (Montpellier, France). Animals were genotyped by PCR using ComR1 primers specific to the floxed region (see Table S1 for primer sequences). Animals were maintained under standard conditions, on a 12:12‐h light/dark schedule and fed chow diet *ad libitum*, according to European Union guidelines for the use of laboratory animals. All experiments were approved by the Ethics Committee for Animal Welfare of the region Languedoc Roussillon (Comité d'Ethique en Expérimentation Animal Languedoc Roussillon), which is an accredited institution of the french “Ministere de l'Enseignement Supérieur, de la Recherche et de l'Innovation” (agreement number D3417227). Animal housing and euthanasia were performed in compliance with the 3 R rule and with recommendations of the Guide for the Care and Use of Laboratory Animals. Experiments were done on 10‐ to 12‐week‐old male and female *mice*.

### 2.10 Histological and immunofluorescence analysis

Immunohistochemistry and immunofluorescence experiments were performed to detect RIP140 and HES1 protein expression. For Immunohistochemistry, tissues from RIP^fl/+^;VilCre, RIP^fl/fl^;VilCre or WT (*i.e*. not expressing the villin‐Cre) mice (see Section 2.9) were fixed with 3.7% paraformaldehyde, embedded in paraffin and sectioned (3 μm). Following incubation in citrate buffer solution, immunohistochemistry analysis was performed using SignalStain^®^ kit according to the manufacturer's instructions. Paraffin‐embedded tissue sections were first incubated in 3% hydrogen peroxide solution to block endogenous peroxidase activity. Each section was then incubated in blocking serum for 3 h to reduce non‐specific binding. Sections were then incubated with antibodies specific for RIP140 (Ab42126, Abcam) and HES1 (#11988, Cell Signaling) diluted in SignalStain^®^ antibody diluent (1 : 100) overnight at 4 °C. Incubation with labeled HRP anti‐rabbit and visualization with diaminobenzidine as a substrate were performed. All slides were counterstained with hematoxylin and images were taken using NanoZoomer (Hamamatsu Photonics, Massy, France).

For immunofluorescence, cells were fixed with 3.7% paraformaldehyde and paraffin‐embedded tissue sections were incubated in citrate buffer solution, then permeabilized with PBS‐1% Triton for 30 min, blocked with PBS 1%BSA for at least 3 h and incubated with the primary antibodies (RIP140, 1 : 100, ab42126; HES1, 1 : 100, #11988) overnight at 4 °C, diluted in PBS 1%BSA. Revelation was performed using Alexa Fluor secondary rabbit antibodies IgG (AF488^®^, AF546^®^, 1/400, Invitrogen^®^). For co‐localization experiment, we used the primary antibody (RIP140, 1 : 100, sc‐9459) and the revelation was made using Alexa Fluor secondary goat antibody IgG (AF488^®^). Slides were counterstained with Hoechst (1/1000, Sigma Aldrich^®^) and mounted with Mowiol (Sigma‐Aldrich^®^) for fluorescence microscopy. Staining quantification was performed at ×40 magnification using the AxioVision software (Carl Zeiss^®^).

### 2.11 TMA construction

As previously described [41], TMA was constructed with FFPE tumor samples (*n* = 45) collected in the frame of the Clinical and Biological Database BCBCOLON (Institut du Cancer de Montpellier – Val d'Aurelle, France, Clinical trial Identifier #NCT03976960). Adenomas, primary adenocarcinomas, and metastatic lesions were sampled as two cores of 1 mm diameter. All samples were chemonaive. Tumor samples were collected from May 2014 to November 2016, following French laws under the supervision of an investigator and declared to the French Ministry of Higher Education and Research (declaration number DC‐2008‐695). The study and its protocols were approved by the French Ethics Committee: CPP (Comité de Protection des Personnes) Sud Méditerrannée III (Ref#2014.02.04) and by the local translational research committee review board (ICM‐CORT‐2018‐28). We have complied with all relevant ethical regulations for work with human participants, and informed written consent was obtained for all patients. This study followed the ethical principles for medical research involving human subjects (WMA Declaration of Helsinki—Ethical Principles for Medical Research Involving Human Subjects—WMA—The World Medical Association).

### 2.12 IHC on human samples

The RIP140 detection has been described previously [30]. HES1 immunostaining of human FFPE CRC samples was performed on a Dako Autostainer using the rabbit monoclonal antibody D6P2U (Cell Signalling Technology). The PT‐Link^®^ system (Dako Agilent, Santa Clara, CA, USA) was used for simultaneous deparaffinization and antigen demasking for 15 min at 95 °C. Endogenous peroxidases and non‐specific signal (Flex Peroxidase Block and Protein Block, Dako) were blocked at room temperature. Sections were incubated for 30 min with the rabbit anti‐human HES1 antibody diluted 1 : 100 in a low background diluent (Dako). A linker rabbit was used to amplify the signal. After two rinses, slides were incubated for 20 min at room temperature with an anti‐rabbit antibody coupled with horseradish peroxidase (HRP) (Flex, Dako). Finally, 3,3'‐diaminobenzidine was used as a substrate for HRP and after rinsing, the slides were counterstained with hematoxylin, dehydrated and mounted with a coverslip.

Two readers performed independently the semiquantitative analysis of immunohistochemical signals. The variations in HES1 or RIP140 expression observed between the different tumors allowed us to establish a score grid (no labeling: 0; weak labeling: 1; moderate labeling: 2; strong labeling: 3). For each sampled spot, the percentage of cells labeled with each intensity was reported. The overall expression was then calculated using the *H*‐Score^2^ method (3 × % of cells with 3+ labeling intensity 2 × % of cells with 2+ intensity and 1 × % of cells with 1+ labeling intensity). The scores obtained thus varied from 0 to 300. To separate the tumors with low or high expression of either RIP140 or HES1, we used a cut‐off corresponding to the median H‐scores. The correlation with patient overall survival was investigated by Kaplan–Meier analysis using the graphpad software and the log rank (Mantel–Cox) test to determine significance.

### 2.13 Cell proliferation

Transient transfected cells were seeded in 96‐well plates (6 replicates per condition), at a density of 5 × 10^3^ cells per well, as previously described [27]. After 4 days, 0.5 mg·mL^−1^ of 3‐(4,5‐dimethylthiazol‐2‐yl)‐2,5‐diphenyltetrazolium bromide (MTT) (Sigma‐Aldrich^®^) was added and incubated at 37 °C for 3 h. Formazan crystals were solubilized in DMSO and absorbance read at 560 nm on a spectrophotometer. Results were normalized to the mean optical density of the control.

Transiently transfected HT29 cells in the absence or presence of the siRNA targeting RIP140 were seeded at a density of 2.5 × 10^3^ cells per well into E‐Plate 16 (ACEA Biosciences, Inc., San Diego, CA, USA) containing 150 μL per well of medium supplemented with 10% FCS. Dynamic monitoring of cell growth was determined every 12 h during 6 days using the impedance‐based xCELLigence system (Roche Applied Science, Penzberg, Germany). The cell index was derived from measured cell‐electrode impedance that correlates with the number of viable cells.

### 2.14 Statistical analyses

All experiments were conducted independently at least three times and the most representative was shown. Statistical analysis was performed using prism software (GraphPad, La Jolla, CA, USA). The statistical significance was evaluated using either Mann–Whitney or Kruskal–Wallis tests or correlation and linear regression. Results were expressed as the mean as mean ± SD. A probability level (*P* value) < 0.05 was considered statistically significant: *P* < 0.05 (*); *P* < 0.01 (**); and *P* < 0.001 (***).

For tumor samples, statistical analysis was performed with the chi‐square or Fisher's exact tests to analyze the associations between categorical variables. All tests were two‐tailed. We used 95% confidence intervals (CIs) and *P* values < 0.05 were considered statistically significant: *P* < 0.05 (*); *P* < 0.01 (**); and *P* < 0.001 (***).

## 3 Results

### 3.1 RIP140 increases the *Hes1* gene expression via an RBPJ/NICD‐mediated mechanism

**Fig. 1:**
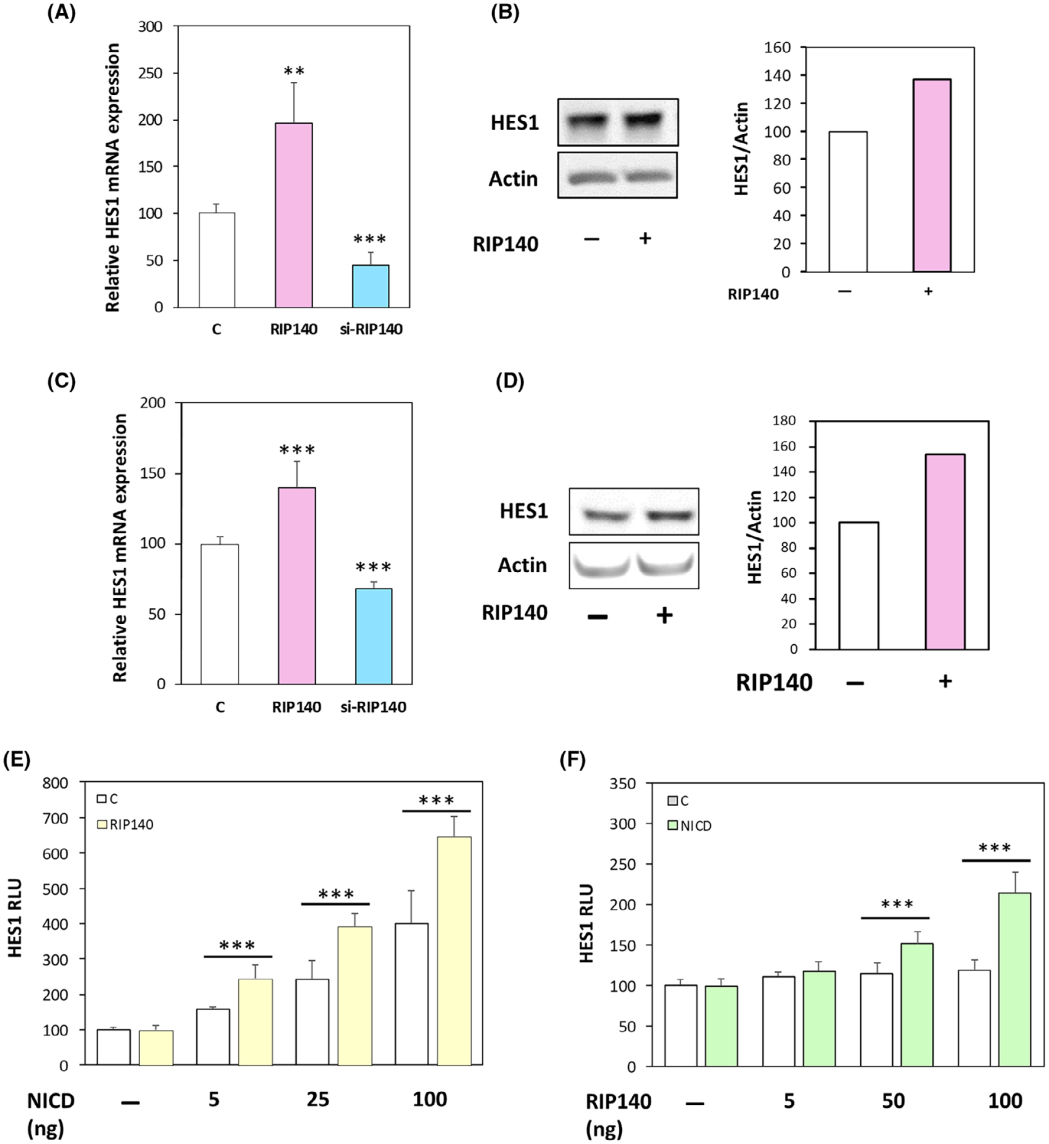
RIP140 increases the HES1 expression at the transcriptional level. (A) *HES1* mRNA level in SW620 CRC cells transiently transfected with control plasmid pEGFP or a control siRNA (C), pEGFP‐RIP140 expression vectors (RIP140) or a RIP140 targeting siRNA (si‐RIP140). Results are expressed as fold change ± standard‐deviation (SD) relatively to controls; *n* = 5 independent experiments for each condition. (B) Western blot analysis of HES1 protein level in SW620 cells transiently transfected or not with a RIP140 expression vector. *n* = 3 independent experiments for each condition. (C) *HES1* mRNA level in HT29 CRC cells transiently transfected as in A. Results are expressed as fold change ± SD relatively to controls; *n* = 4 independent experiments for each condition. (D) Western blot analysis of HES1 protein level in HT29 cells transiently transfected with the RIP140 expression vector. *n* = 3 independent experiments for each condition. (E) Luciferase reporter assay performed with the reporter construct encompassing the 2.5 kb promoter region of *HES1* gene was transiently co‐transfected into SW620 CRC cells with increasing doses of NICD expression vector in the presence (RIP140) or not (C) of the RIP140 expression vector. Relative luciferase unit (RLU) was expressed as mean ± SD; *n* = 3 independent experiments. (F) A reporter construct encompassing the promoter region of the *HES1* gene (0.47 kb) was transiently co‐transfected into SW620 cells with increasing doses of RIP140 expression vector in the presence or not of NICD expression vector; *n* = 3 independent experiments. For all panels: **P < 0.01 and ***P < 0.001 (Mann–Whitney test).

To decipher the role of RIP140 on the Notch/HES1 pathway, we first measured its effect on *HES1* gene expression in different CRC cell lines in normal culture conditions. As shown in Fig. 1A, ectopic expression of RIP140 in SW620 CRC cells significantly increased the level of *Hes1* mRNA, whereas the opposite effect was observed upon *Rip140* knock‐down after transfection of a specific siRNA. The positive effect of RIP140 was also noticed on HES1 protein levels assessed by the western‐blot analysis (Fig. 1B). Moreover, the same regulation of *Hes1* mRNA and protein levels was obtained in HT29 CRC cells (Fig. 1C,D).

To further investigate the mechanisms involved in this regulation, we transiently transfected a luciferase reporter vector containing the promoter region of the *HES1* gene (encompassing the sequence from −2615 to +46) [36] together with increasing doses of RIP140 expression vector. As shown in Fig. 1E, we observed a dose‐dependent transactivation by RIP140. In addition, we performed the same experiment in the presence or absence of a NICD expressing plasmid (Fig. 1F) and found that the transactivation induced by the NICD‐encoding plasmid was significantly increased by in the presence of the RIP140 expression vector. Altogether, these data clearly demonstrated that RIP140 exerted a NICD‐dependent positive transcriptional regulation of *HES1* gene expression in human CRC cells.

**Fig. 2:**
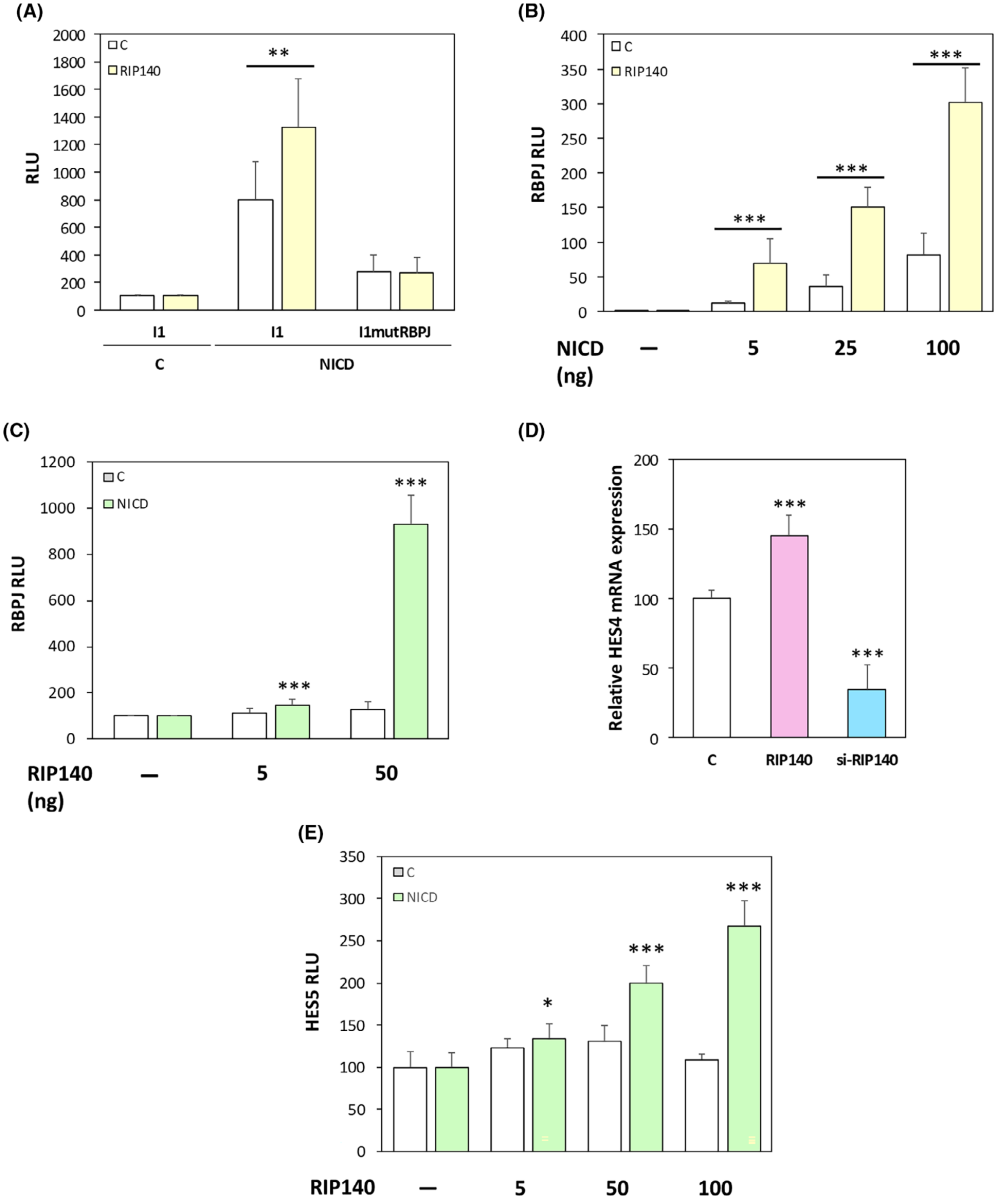
RIP140 regulates HES1 via an RBPJ/NICD mechanism. (A) The very proximal promoter region of HES1 with the RBPJ binding site and a remaining HES1 binding site (I1 construct) and I1 lacking the RBPJ site (I1mutRBPJ construct) were transiently co‐transfected into SW620 cells with doses of NICD and RIP140 expression vectors. Results are expressed as fold change ± standard deviation (SD) relatively to controls; *n* = 3 independent experiments. (B) Luciferase reporter assay performed on an artificial reporter construct containing 6 RBPJ‐binding sites in the same conditions as in panel C of Fig. 1. Relative luciferase unit (RLU) was expressed as mean ± SD; *n* = 3 independent experiments. (C) Luciferase reporter assay performed on an artificial reporter construct containing 6 RBPJ‐binding sites in the same conditions as in panel (B). Results are expressed as fold change ± SD relatively to controls; *n* = 3 independent experiments. (D) *HES4* mRNA level in SW620 cells measured under the same condition as in panel A. Results are expressed as fold change ± SD relatively to controls; *n* = 3 independent experiments for each condition. (E) A reporter construct encompassing the *HES5* gene promoter was co‐transfected into SW620 cells with increasing doses of RIP140 expression vector in the presence or not of NICD expression vector. Results are expressed as fold change ± SD relatively to controls; *n* = 3 independent experiments. For all panels: *P < 0.05, **P < 0.01, and ***P < 0.001 (Mann–Whitney test).

To decipher further the underlying mechanisms, we generated several mutant versions of the *HES1* luciferase reporter construct. As shown in Fig. 2A, the I1 plasmid which contains only the proximal promoter region of the *HES1* gene (−125 to +46) supported a positive NICD‐mediated effect of RIP140. This NICD‐dependent positive transcriptional regulation of the *HES1* gene by RIP140 was totally abolished upon mutagenesis of the RBPJ binding site, thus further demonstrating that the effect of RIP140 on *HES1* gene expression was RBPJ/NICD‐mediated. This was confirmed by the use of an artificial reporter construct, which contains several RBPJ response elements upstream the minimal promoter driving the luciferase. Using this simplified RBPJ‐Luciferase reporter plasmid, we obtained a significant stronger amplification of NICD‐mediated transactivation by RIP140 both in SW620 (Fig. 2B) and in HT29 CRC cells. Moreover, as above‐mentioned for the *HES1* gene reporter (Fig. 1D), the dose‐dependent positive effect of RIP140 on the RBPJ‐Luciferase reporter plasmid was observed only in the presence of NICD (Fig. 2C). Finally, in accordance with these observations, we validate the NICD‐mediated positive effect of RIP140 on *HES4* mRNA expression (Fig. 2D) and on *HES5* gene transcription (Fig. 2E), both genes being also Notch target genes [42, 43]. Altogether, these data demonstrated that, in human CRC cells, RIP140 increases the Notch pathway as demonstrated by the induction of RBPJ/NICD‐mediated transcription of several targets of the Notch signaling, including the *HES1* gene.

### 3.2 RIP140 is a HES1 target gene

**Fig. 3:**
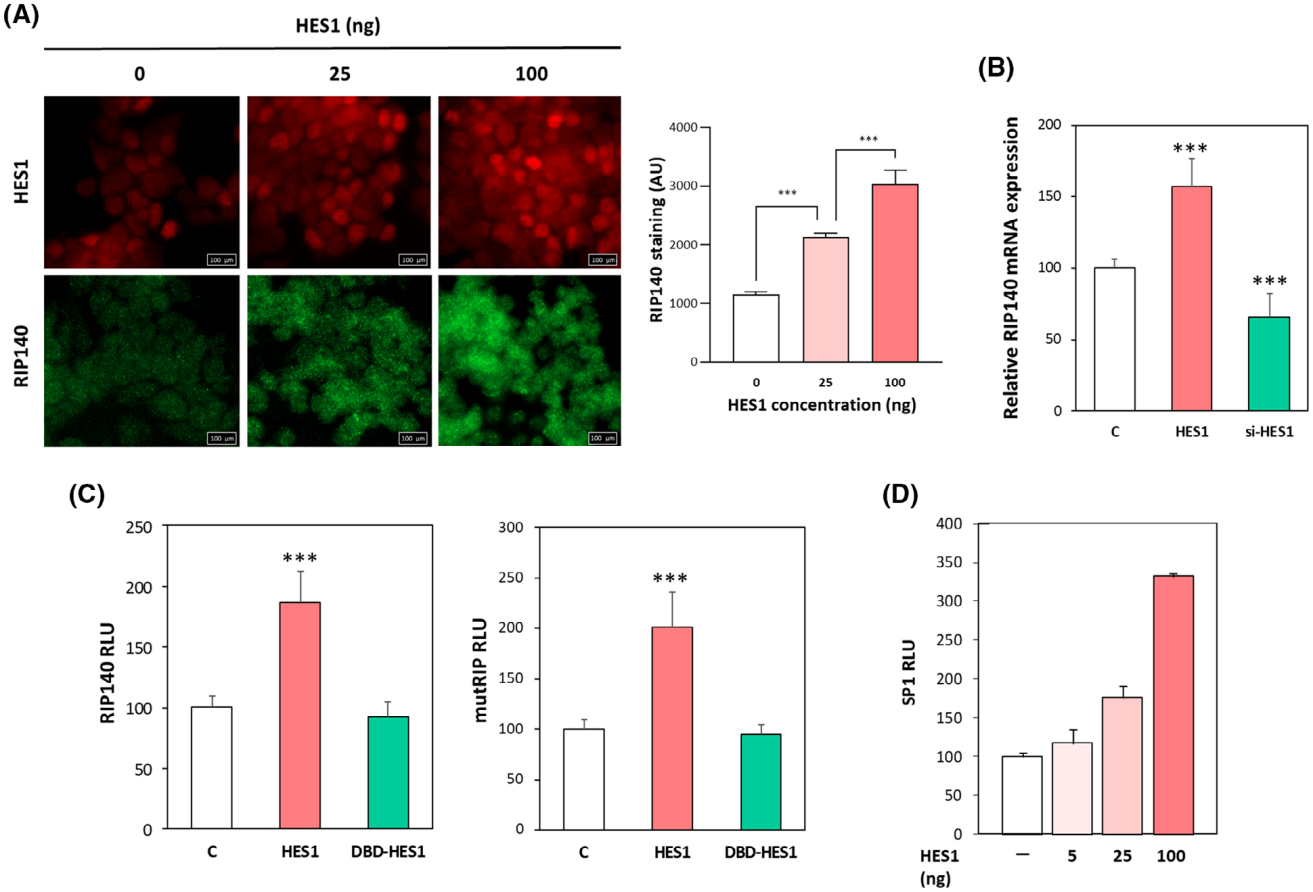
RIP140 gene expression is increased by HES1. (A) Immunofluorescence analysis (40×) of HES1 and RIP140 protein levels in SW620 cells transiently transfected with increasing doses of HES1 expression vector (25 and 100 ng). Results of the staining quantification are expressed as fold change ± standard‐deviation (SD) relatively to controls; *n* = 6 fields per condition. Scale bar: 50 μm. (B) *RIP140* mRNA level in SW620 cells transiently transfected or not with HES1 or with a siRNA targeting the *HES1* mRNA (si‐HES1). Results are expressed as fold change ± SD relatively to the control; *n* = 4 independent experiments. (C) Luciferase reporter assay performed on an artificial reporter construct containing either the promoter region of the human *RIP140* gene encompassing the sequence from −814 to +106 (left panels) or a more proximal sequence from −158 to +106 (right panel) were transiently co‐transfected into SW620 cells with HES1 or DBD‐HES1 expression vectors. Relative luciferase unit (RLU) was expressed as mean ± SD; *n* = 3 independent experiments. (D) An artificial reporter construct encompassing three SP1 binding sites was transiently co‐transfected into SW620 cells with increases doses of HES1 expression vectors. Relative luciferase unit (RLU) was expressed as mean ± SD; *n* = 3 independent experiments. For all panels: ***P < 0.001 (Mann–Whitney test).

As observed for other transcription factors engaged in negative feedback loop with RIP140 including for instance the estrogen receptor [15] or the E2F1 transcription factor [17, 39], we expected a possible induction of the *RIP140* gene transcription by HES1. As shown in Fig. 3A, HES1 ectopic expression indeed significantly increased the accumulation of the RIP140 protein detected by immunofluorescence. The same regulation of *RIP140* gene expression was observed at the mRNA level upon HES1 ectopic expression and the opposite effect was obtained upon HES1 knock‐down using a specific siRNA in SW620 cells (Fig. 3B) and similar results were obtained in HT29 cells (Fig. S1A,B).

Interestingly, this regulation of RIP140 expression by the HES1 pathway was noticed at the transcriptional level upon HES1 ectopic expression (Fig. 3C). These data were obtained on a luciferase reporter construct containing either the promoter region of the human *RIP140* gene encompassing the sequence from −814 to +106 (left panels) or a more proximal sequence from −158 to +106 (right panels) [32]. As expected, the DBD mutated version of HES1 was unable to increase *RIP140* gene transcription. It should be noted that the same opposite effects were measured in HT29 CRC cells (Fig. S1C) and observed, at the transcriptional level, on the murine *Rip140* promoter, in both SW620 and HT29 CRC cells (Fig. S1D). Moreover, a similar positive effect upon HES1 ectopic expression was observed on a reporter construct encompassing the luciferase gene controlled by three Sp1 binding sites [38] (Fig. 3D). This suggests that the regulation of *RIP140* gene expression by HES1 is possibly Sp1‐mediated.

### 3.3 Correlation between the RIP140 and HES1 expression in mouse and human tissues

The above‐mentioned molecular analyses performed in human cancer cell lines unraveled positive transcriptional regulatory links between RIP140 and HES1. In order to assess the relative expression of the two transcription factors in more physiological conditions, we analyzed their expression at the mRNA and protein levels in mouse tissue samples.

**Fig. 4:**
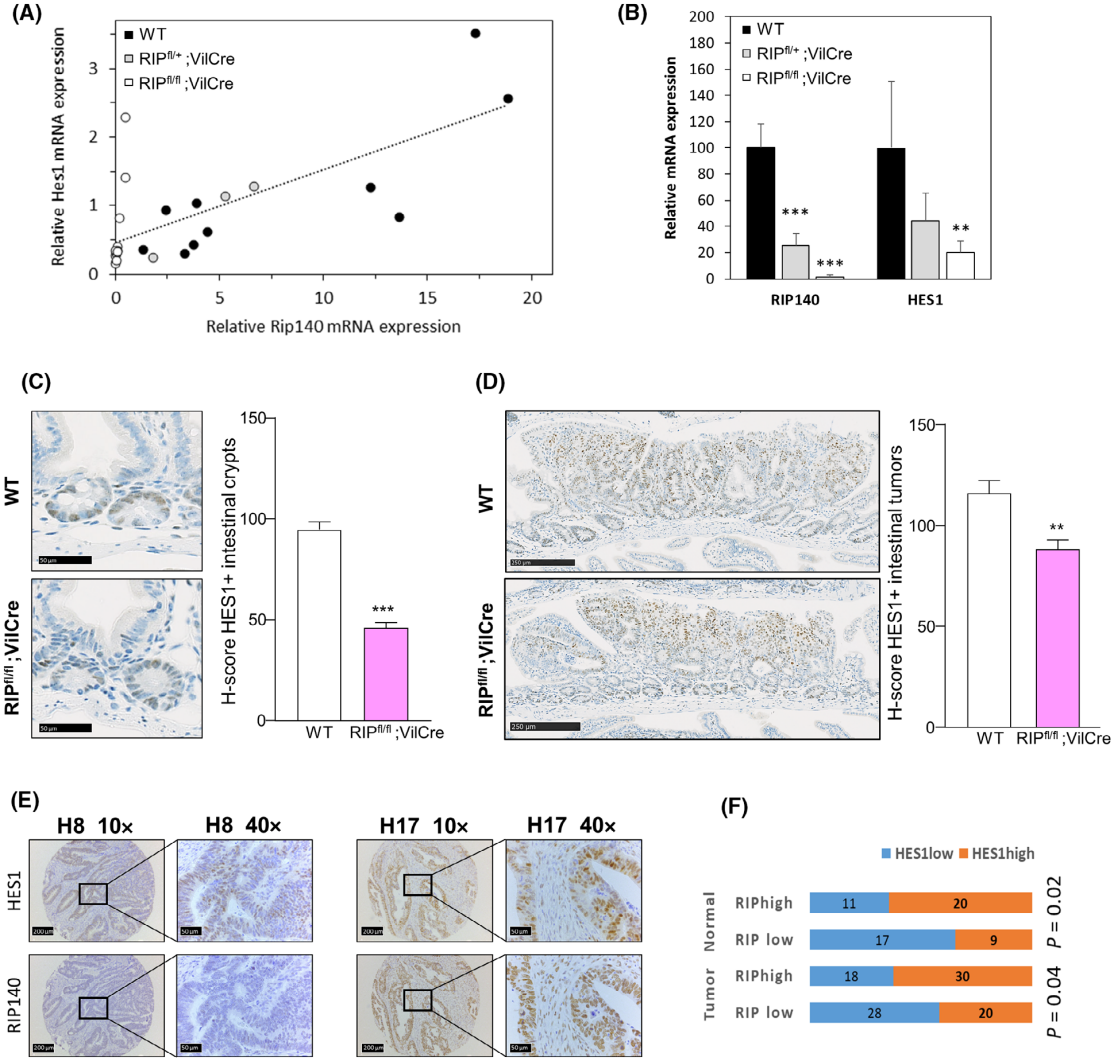
Positive correlation between RIP140 and HES1 expression in mouse intestine tissues and in human CRCs. (A) Correlation between *Rip140* and *Hes1* gene expression in intestinal epithelium cell fraction of conditional RIP140 knock‐out (KO) mice (RIP^fl/+^;VilCre and RIP^fl/fl^;VilCre in gray and white circles respectively; *n* = 3 and 9 mice respectively) compared to their wild‐type littermates (WT; in black circles, *n* = 9 mice). The dotted line represents the linear regression line to model the positive correlation between the expression of the two variables, RIP140 and HES1. (B) *Rip140* and *Hes1* gene expression in intestinal epithelium cells fraction of *Rip140* conditional KO mice (RIP^fl/+^;VilCre and RIP^fl/fl^;VilCre) compared to their wild‐type littermates (RIP^fl/+^;VilCre^+/+^;WT). Results are expressed as fold change ± standard‐deviation (S.D). relatively to the control; *n* = 3, 9 and 9 mice respectively. (C) Immunohistochemistry and quantification ± SD of HES1 staining (*H*‐score values) in normal intestinal crypts of RIP^fl/fl^;VilCre mice compared to their wild‐type littermates (WT). Scale bar: 50 μm. *n* = 6 mice per genotype. (D) Same as in (C) in intestinal tumors from RIP^fl/fl^;VilCre mice compared to their littermates (WT). Scale bar: 250 μm. *n* = 6 mice per genotype. (E) Example of HES1 and RIP140 IHC staining performed as described in Section 2 and illustrating low (left panels) or high (right panels) intensity staining. The tissue sample name and the tumor magnification are indicated above the photographs. Scale bars: 200 and 50 μm. (F) Schematic representation of the distribution of tumors with low or high HES1 expression (median cut‐off) in the two subgroups of CRC with low (RIPlow) or high (RIPhigh) RIP140 expression (median cut‐off). Statistical analysis was performed using the *Chi‐2* test and *P*‐values are indicated. For all panels: **P < 0.01 and ***P < 0.001 (Mann–Whitney test).

We used transgenic mice exhibiting a specific invalidation of the *Rip140* gene in the intestinal epithelium (RIPcKO^int^). These animals were obtained by crossing mice bearing the floxed *Rip140* gene with a strain expressing the Cre recombinase under the control of the *Villin* gene promoter [40]. As shown in Fig. 4A,B, we observed a strong correlation between *Rip140* and *Hes1* mRNA levels (*r* = 0.506; *P* < 0.0005). The lowest levels of *Hes1* mRNA were measured in the intestinal epithelium of mice with the homozygous deletion of the *Rip140* gene as compared to wild‐type mice (*Hes1* mRNA levels being intermediate in mice with heterozygous deletion of the *Rip140* gene). These results were validated at the protein level by immunohistochemistry in tissue sections of intestine from the RIPcKO^int^ compared to wild‐type animals. The nuclear HES1 staining observed in the intestinal crypts was significantly decreased in the epithelium of RIPKO mice (Fig. 4C). In line with this observation and our previous result, we also found a decrease of HES1 staining in the intestinal tumors developed by the RIPcKO^int^ mice crossed with the tumor‐prone *Apc* ^flox^ mouse strain, as compared to the control animals (Fig. 4D).

**Fig. 5:**
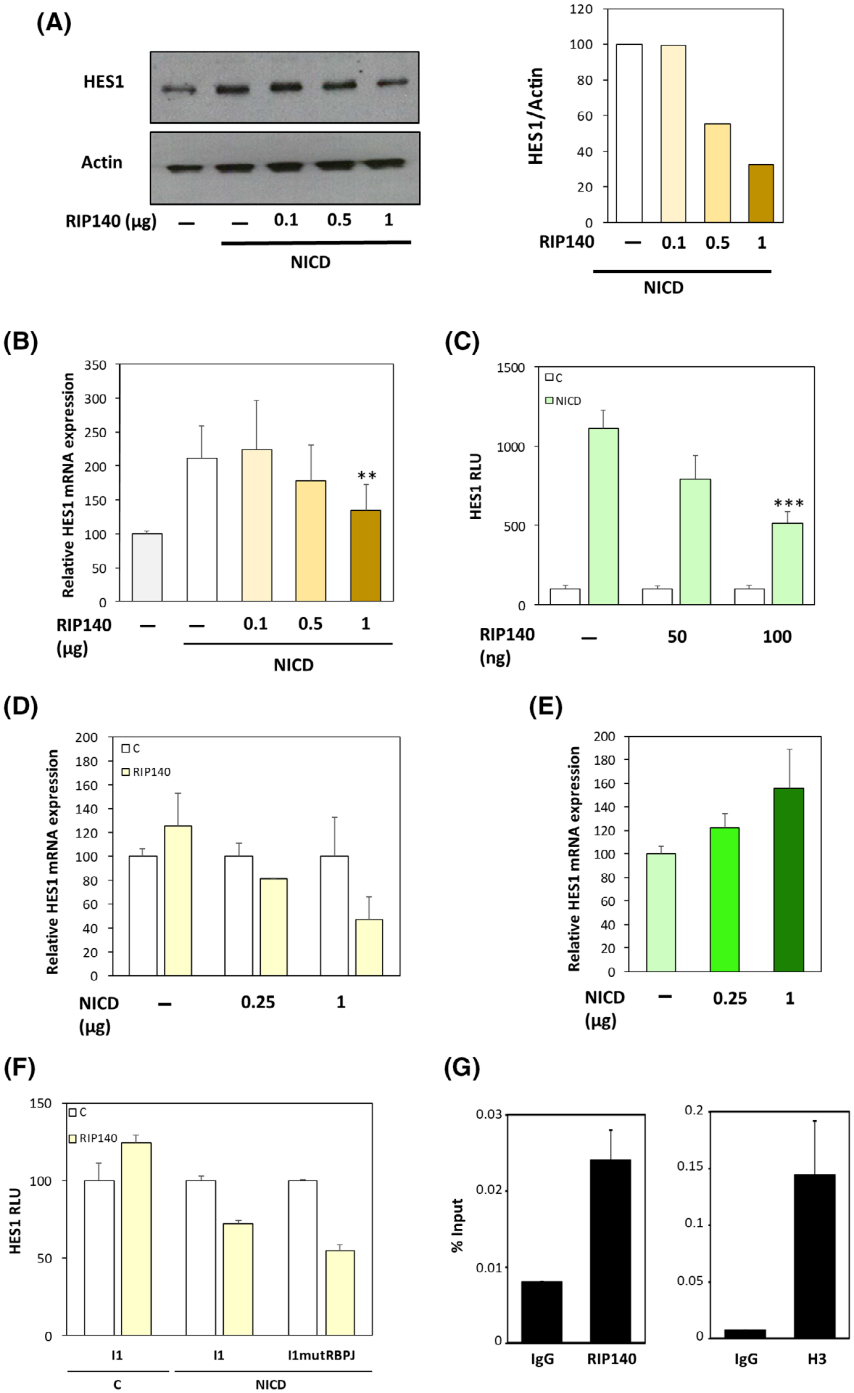
RIP140 inhibits HES1 gene expression when it is highly expressed. (A) Western blot analysis of HES1 protein level in SW620 cells transiently transfected with increasing doses of RIP140 expression vector (0.1, 0.5 and 1 μg) and a high dose of NICD expression vector (1 μg). *n* = 3 independent experiments for each condition. (B) *HES1* mRNA level in SW620 cells measured under the same conditions as in panel (A). Results are expressed as fold change ± standard deviation (SD) relatively to the control *n* = 3 independent experiments. (C) Luciferase reporter assay performed on the *HES1* gene promoter (0.47 kb) construct in SW620 cells transiently co‐transfected with increasing doses of RIP140 expression vector in the presence or not of NICD expression vector. Relative luciferase unit (RLU) was expressed as mean ± SD; *n* = 3 independent experiments. (D) RT‐qPCR analysis of *HES1* mRNA levels in SW620 cells transiently transfected with increasing doses of NICD expression vector in the presence (RIP140) or not (−) of RIP140 expression vector. Results are expressed as fold change ± SD relatively to the control; *n* = 3 independent experiments. (E) Quantification of HES1 mRNA levels in the different NICD conditions shown in panel (D) without ectopic expression of RIP140. Results are expressed as fold change ± SD relatively to the control; *n* = 3 independent experiments. (F) I1 and I1mutRBPJ reporter constructs were transiently co‐transfected into SW620 cells in the presence or not of NICD and/or RIP140 expression vectors. Relative luciferase unit (RLU) was expressed as mean ± SD; *n* = 3 independent experiments. (G) ChIP assay using HT29 cells and anti‐IgG, anti‐RIP140 or anti‐H3pan antibodies. Purified DNA was amplified by qPCR using HES1 promoter primer pairs. Results are expressed as fold change ± SD relatively to the input; *n* = 3 independent experiments. For all panels: **P < 0.01 and ***P < 0.001 (Mann–Whitney test).

To validate these data in human tissues, we monitored the expression of RIP140 and HES1 proteins by immunohistochemistry in a cohort of human CRC biopsies. This cohort comprised 45 primary tumors of different stages (7 adenomas, 9 stage I, 10 stage II, 12 stage III, 7 stage IV) and 9 metastases (see Table 1 for patient characteristics). The expressions of RIP140 and HES1 were quantified both in the non‐tumoral mucosa and in the adenocarcinoma, as described in Section 2. As shown in Fig. 5E,F, the nuclear staining of the two markers was significantly correlated in both normal and tumoral intestinal tissues. It should be noted that a positive and significant correlation between RIP140 and HES1 expression was also noticed at the mRNA level in primary colorectal tumors and in normal adjacent tissues after reanalysis of the *Colonomics* dataset using the *Cancertool* database [44] (Fig. S2).

**Table 1:** Characteristics of the CRC patient cohort (*n* = 45).

	*n*	%
Gender	41	
Male	25	61%
Female	16	39%
Age at first diagnosis (years)		
Median	67	
[Min–Max]	[51–82]	
Samples	48	
Localization of the tumor		
Right colon	16	33%
Transverse colon	6	13%
Left colon	15	31%
Colon (no specific information)	3	6%
Liver	6	13%
Peritoneum	2	4%
Stage		
Adenoma	5	10%
Primary tumor—Stage I	7	15%
Primary tumor—Stage II	9	19%
Primary tumor—Stage III	11	23%
Primary tumor—Stage IV	8	17%
Metastasis	8	17%
RAS status		
WT	24	50%
Mutated	24	50%
BRAF status		
WT	48	100%
Mutated	0	0%
Microsatellite status		
MSS	43	90%
MSI	5	10%

### 3.4 Repressive effect of RIP140 when HES1 is expressed at high levels

As described above, our data evidenced a clear positive transcriptional regulation of the *HES1* gene by RIP140. However, the role of RIP140 in the control of *HES1* gene expression appeared more complex since we noticed in a reproducible way that, in conditions where HES1 expression was strongly induced by NICD, RIP140 exerted an inhibition of *HES1* gene transcription. Indeed, in such conditions, RIP140 behaved clearly as an inhibitor of *HES1* gene expression, as shown at the protein (Fig. 5A), mRNA (Fig. 5B) and transcriptional level (Fig. 5C). Again, similar results were obtained in HT29 CRC cells (Fig. S3A,B) and on the *HES4* mRNA expression (Fig. S3C), thus demonstrating that the negative regulation exerted by RIP140, when the Notch pathway was fully activated, was a common effect.

As shown in Fig. 5D, the switch in the effect of RIP140 from a positive to a negative regulation was clearly associated with the NICD induction of *HES1* gene expression (Fig. 5E). The same switch from an activator to a repressor, according to NICD ectopic expression, was observed in HT29 cells at the transcriptional level on the *HES1* reporter construct (Fig. S3D). The inhibitory effect of RIP140 was also detected on the I1 construct encompassing the *HES1* proximal promoter (Fig. 5F). More importantly, the negative effect of RIP140, which occurred at high NICD levels, was not abolished by the mutation of the RBPJ binding site (Fig. 5F).

Finally, ChIP experiments (Fig. 5G) demonstrated the specific recruitment of NRIP1 to the *HES1* promoter sequence that encompassed the RBPJ and HES1 binding sites that conferred regulation by NRIP1 in the above‐mentioned luciferase assays. The amplification signal was lower than that observed after anti‐histone H3 ChIP (H3) but higher than with control IgG. Moreover, we did not observe any recruitment to an irrelevant region.

Collectively, these data strongly suggested that, depending on HES1 protein level, RIP140 behaved as a direct activator or a repressor of *HES1* gene transcription.

### 3.5 RIP140 is involved in the HES1 negative transcription feedback loop

The oscillatory expression of the *HES1* gene and its regulation by a negative feedback loop have been reported more than 15 years ago [45]. One hypothesis that might recapitulate the effects of RIP140 on *HES1* gene expression relies on the participation of RIP140 in this HES1 negative feedback loop.

To validate this hypothesis, we set up an assay allowing the quantification of the endogenous *HES1* gene expression in the presence or not of ectopic expression of the HES1 and/or RIP140 proteins. As shown in Fig. S4A (top scheme), we designed a specific RT‐qPCR based quantification of the endogenous *HES1* gene expression by using specific primers in the 3'UTR region of the HES1 mRNA. Upon HES1 ectopic expression monitored using primers located in the coding sequence of the gene and allowing quantification of both ectopic and endogenous HES1 mRNAs (Fig. S4A – left panel), we observed the previously described negative feedback loop [45] leading to a decreased endogenous HES1 RNA level (right panel).

**Fig. 6:**
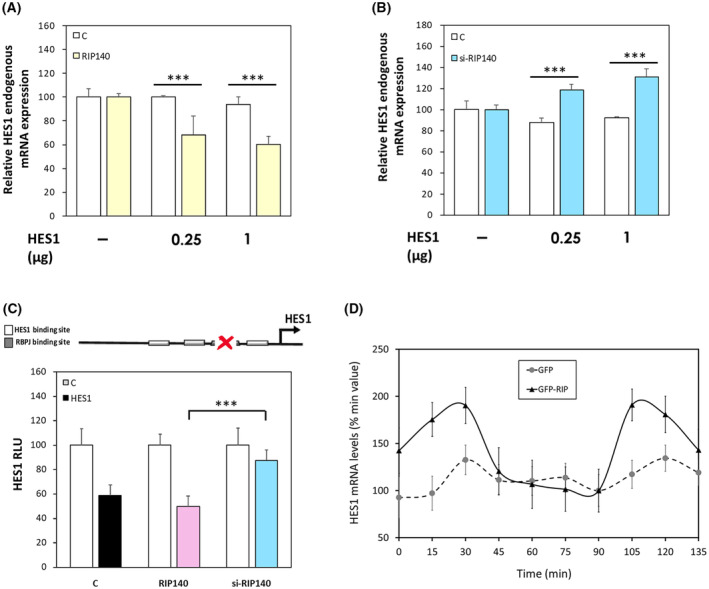
RIP140 is involved in the HES1 feedback loop. (A) *HES1* endogenous gene expression in SW620 cells transiently transfected with increasing doses of HES1 expression vector in the presence or not of RIP140 expression vector. Results are expressed as fold change ± standard‐deviation (SD) relatively to the control; *n* = 3 independent experiments. (B) *HES1* endogenous gene expression in SW620 cells transiently co‐transfected with increased doses of HES1 expression vector together with a control siRNA (−) or a siRNA targeting RIP140 (si‐RIP140). Results are expressed as fold change ± SD relatively to the control; *n* = 3 independent experiments. (C) The *HES1* gene promoter construct (I3mutRBPJ construct) containing the three HES1 binding sites and the mutated RBPJ binding site was transiently co‐transfected in SW620 cells. The RIP140 expression vector or a siRNA targeting RIP140 (si‐RIP140) together with the HES1 expression vector was co‐transfected or not. Relative luciferase unit (RLU) was expressed as mean ± SD; *n* = 3 independent experiments. (D) Oscillatory expression of HES1 mRNA in SW620 cells transiently transfected with pEGFP (GFP) or pEGFP‐RIP140 expression vectors (GFP‐RIP140). Results are expressed as fold change ± SD relatively to a minimum value; *n* = 3 independent experiments for each condition. For all panels: ***P < 0.001 (Mann–Whitney test).

Using this assay, we confirmed that the negative effect of RIP140 on endogenous *HES1* mRNA levels was indeed dependent on HES1 ectopic expression (Fig. 6A). More importantly, we demonstrated that RIP140 was required for the HES1 negative feedback‐loop since the negative effect of HES1 on its own expression was abolished (and even reversed) upon siRNA silencing of *RIP140* gene expression (Fig. 6B). The reinforcement by RIP140 of the inhibitory effect of HES1 on its own expression was also observed at the transcriptional level using the I3mutRBPJ reporter construct containing the HES1 promoter region mutated on the RBPJ binding sites and thus bearing only the HES1 response elements (Fig. 6C). The importance of RIP140 in the inhibitory effect of HES1 was confirmed at the transcriptional level upon siRNA silencing of *RIP140* gene expression (Fig. 6C). Finally, these results were strengthened by data showing that ectopic expression of RIP140 in SW620 cells increased the oscillatory expression of the HES1 mRNA as measured by immunofluorescence (Fig. 6D).

**Fig. 7:**
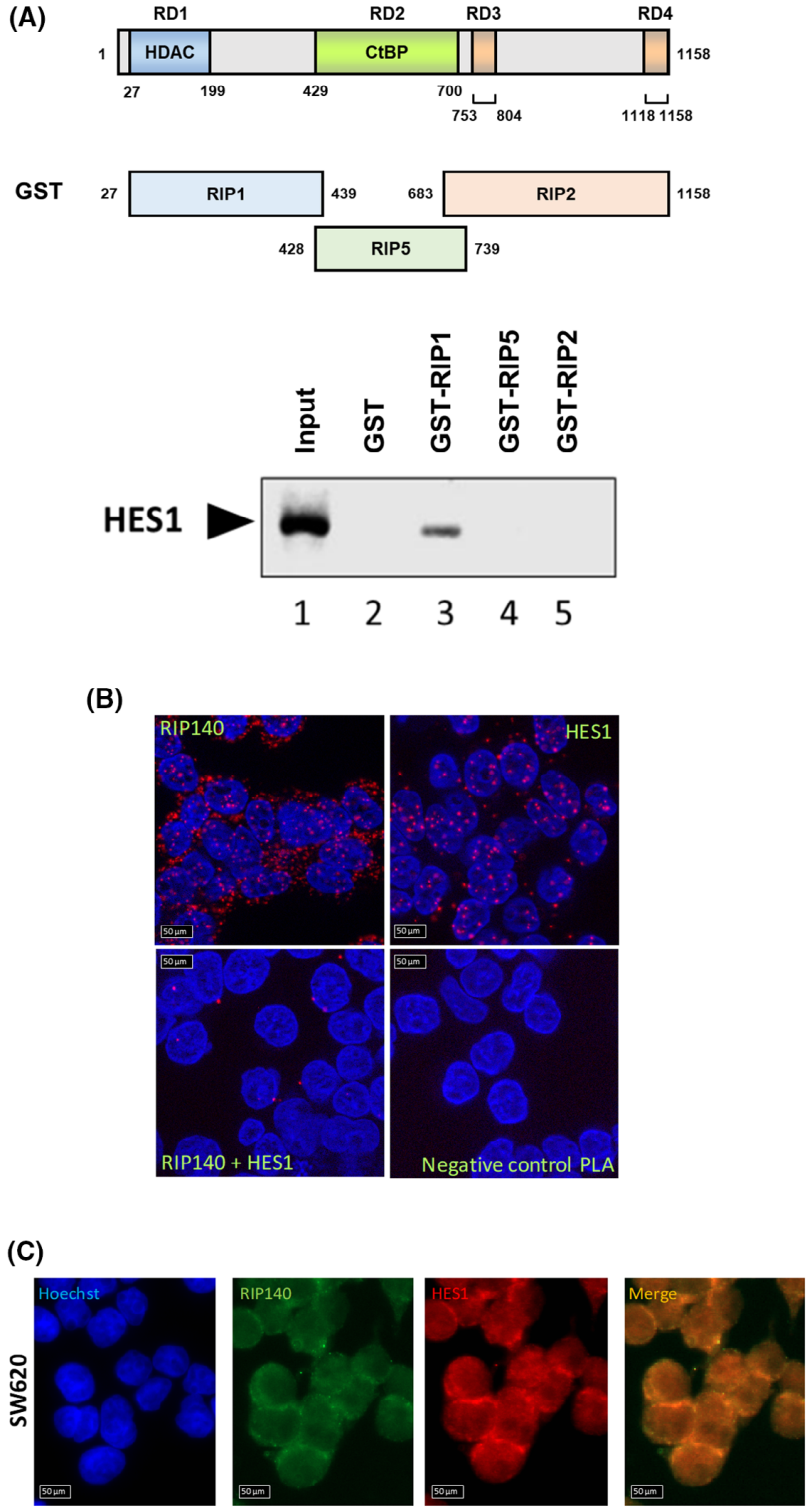
RIP140 interacts with HES1. (A) Analysis of RIP140 *in vitro* interaction with HES1 protein by GST pull down. The HES1 protein input is shown in lane1. The figure is representative of two independent experiments. (B) DuoLink proximity ligation assay performed to visualized endogenous HES1 and RIP140 interaction in SW620 cells. Scale bar: 50 μm. The figure is representative of three independent experiments. (C) Double immunofluorescence analysis of HES1 and RIP140 protein levels in SW620 cells. Scale bar: 50 μm. The figure is representative of three independent experiments.

To further characterize the effect of RIP140 on HES1 repressive activity, we investigated whether the two proteins were able to interact. As shown in Fig. 7A, GST pull‐down experiments clearly demonstrated that the N‐terminal region (fragment RIP1 that encompasses the RIP140 sequence from amino acid 27 to 439) was able to bind to the *in vitro* translated HES1 protein. The interaction between the two endogenous proteins was confirmed by proximity ligation assay (Fig. 7B) and co‐localization experiment in intact SW620 CRC cells (Fig. 7C). Similar results were obtained in HT29 CRC cells (Fig. S4C,D). Altogether, these data supported the conclusion that RIP140 interacts with HES1 and is required for its full repressive activity, in particular on its own promoter.

### 3.6 RIP140 switches the mitogenic effect of HES1 on CRC cell proliferation

**Fig. 8:**
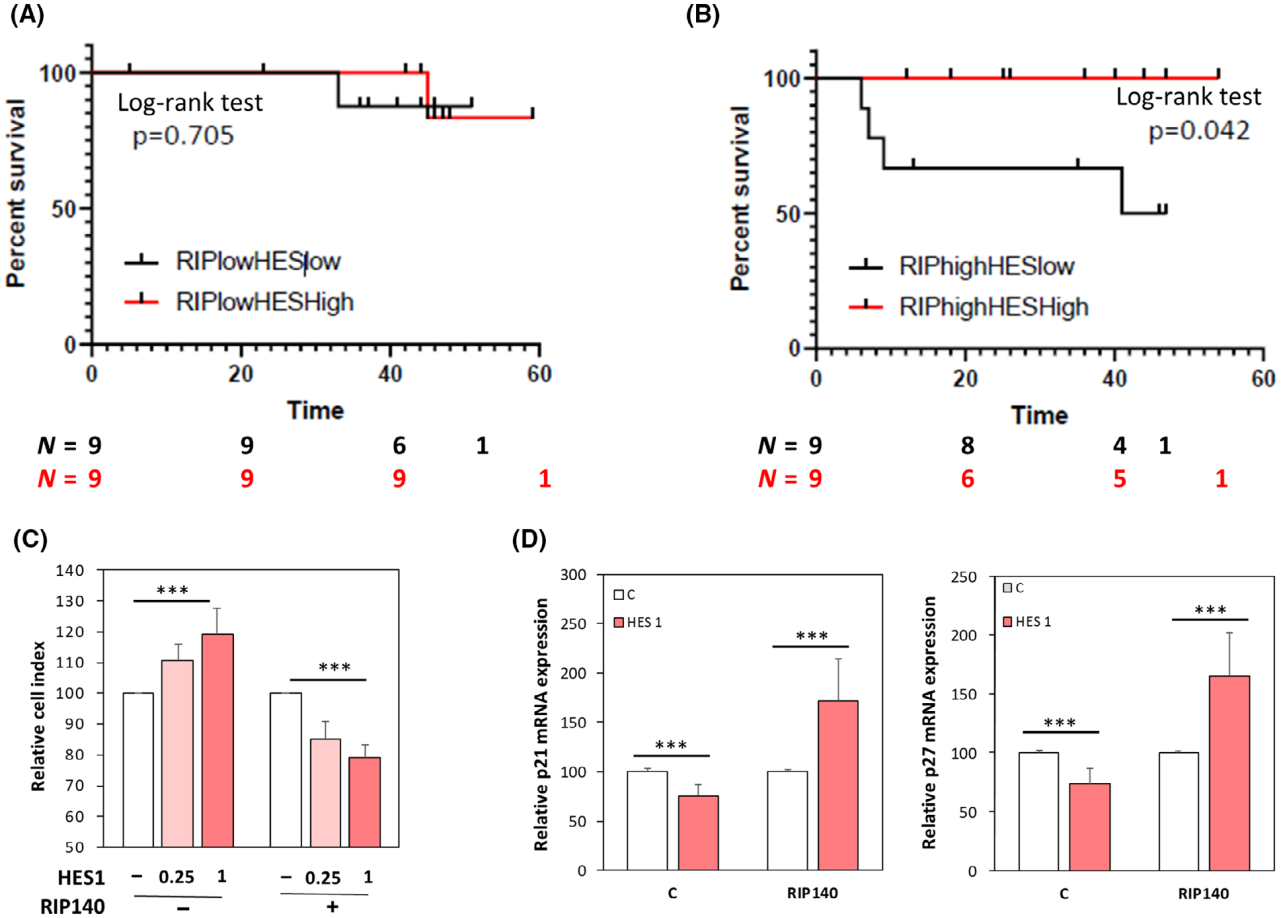
Crosstalk between RIP140 and HES1 in human CRC. (A) and (B) Kaplan–Meier analysis of the cumulative overall survival (OS) of patients with low or high HES1 staining immunoreactive score (IRS) in their tumors was performed on the groups exhibiting low (panel A) or high (panel B) RIP140 staining IRS (best cut‐off threshold). A log‐rank test was used for the statistical analysis. The number of patient at risk in each condition has been added under the curves. (C) The proliferation of SW620 cells transiently transfected with increasing doses of HES1 expression vector in the presence or not of RIP140 expression vector was quantified at day 4 using an MTT assay. Results represent the fold change ± standard‐deviation (SD) vs. levels in control cells; *n* = 2 independent experiments. (D) *p21* (left panel) and *p27* (right panel) mRNA levels in HT29 cells transiently transfected with RIP140 and HES1 expression vectors. Results are expressed as fold change ± SD relatively to control; *n* = 3 independent experiments. For all panels: ***P < 0.001 (Mann–Whitney test).

We then investigated whether this intimate cross‐talk between the two transcription factors might be relevant in CRC pathogenesis. We first performed Kaplan–Meier analyses of CRC patient survival based on HES1 expression. In the whole cohort, HES1 levels were not significantly associated with patient survival (Fig. S5A). Same results were obtained when we analyzed the tumors with the lowest level of RIP140 based on median cut‐off (Fig. 8A). In contrast, as shown in Fig. 8B, high level of HES1 and RIP140 was associated with an increased patient survival.

These IHC data were confirmed at the level of mRNA expression by reanalyzing transcriptomic data from the TCGA (http://tcga‐data.nci.nih.gov/docs/publications/coadread_2012/) [46]. Using the Kaplan–Meier plotter database, we reanalyzed RNAseq data obtained on colon adenocarcinoma from 452 patients. This cohort was separated into two groups of 226 patients with low and high RIP140 expression in the corresponding tumors using the median as a cutoff value (Fig. S5B,C, respectively). As observed by IHC and despite a very low number of samples in each arms, we confirmed a statistically significant association of high expression of HES1 with a decreased risk of death in colon cancer patients, only when their tumor express high RIP140 expression (Fig. S5C, *P* = 0.048). Altogether, the data strongly suggested that RIP140 influences the biological activity of the HES1 protein, in accordance with our data demonstrating a strong effect of RIP140 on HES1 expression and transcriptional activity.

We then attempted to demonstrate experimentally this impact of RIP140 on HES1 oncogenic activity. As shown in Fig. S5D, we first validated that RIP140 exerted a clear antiproliferative activity in CRC cells since its knockdown in human CRC cells produced a significant mitogenic effect. These data confirmed previous results obtained in the HCT116 cell line [27] and were validated in other CRC cell lines. Very interestingly, when we tested the simultaneous ectopic expression of HES1 and RIP140, we clearly observed that overexpression of RIP140 switched HES1 effect from a stimulation to an inhibition of CRC cell proliferation (Fig. 8C). Similar results were obtained on the expression of the *p21* and *p27* genes (Fig. 8D). These two cyclin‐dependent kinase inhibitors are key regulators of cell proliferation and survival which induce cell cycle arrest by inhibiting the activity of several cyclin‐dependent kinases [47]. These data strengthened the major switch exerted by RIP140 on the regulation of CRC cell proliferation by HES1 and explained, at least partly, why HES1 has a good prognosis value in CRC with high levels of RIP140.

## 4 Discussion

**Fig. 9:**
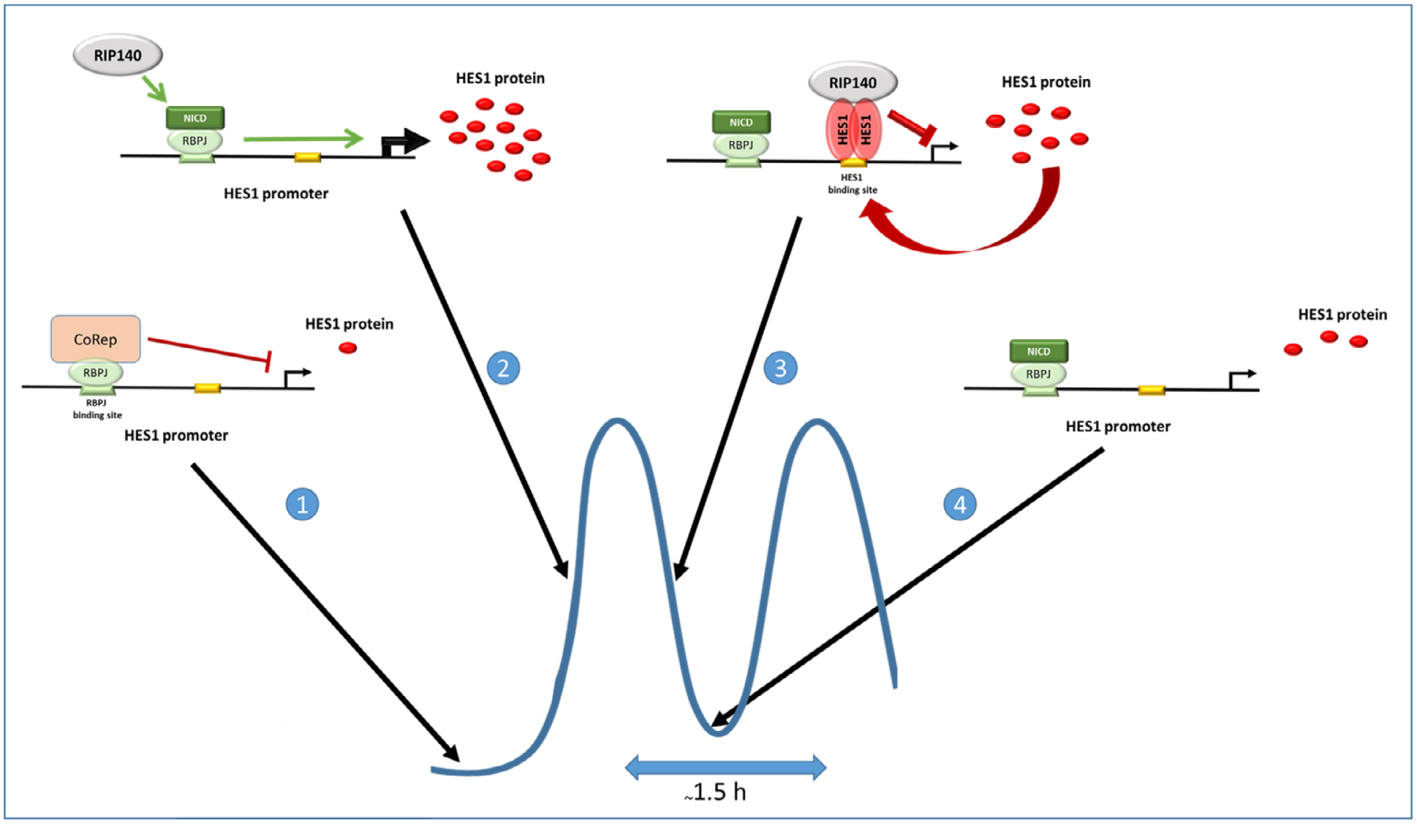
Schematic representation of the role of RIP140 in the regulation of HES1 expression in CRC cells. (1) In the absence of Notch activation, there is a low level of *HES1* gene transcription because RBPJ is engaged in a repressive complex. (2) Upon Notch pathway activation, NICD is translocated to the nucleus and binds RBPJ to activate *HES1* gene transcription with the help of RIP140, which might act indirectly (*i.e*. by titrating corepressors away from RBPJ). (3) Once the level of the HES1 protein reaches a certain threshold, it binds its own promoter and recruits RIP40 through direct protein–protein interaction in order to repress its gene transcription. (4) Due the rapid turn‐over of the HES1 protein, this negative feedback loop disappears very quickly leaving the re‐apparition of step 2 and another cycle of induction of HES1 transcription (one cycle during roughly 1.5 h).

Colorectal cancer is a frequent neoplasm that implicates the deregulation of multiple signaling pathways involved in the control of intestinal epithelial cell differentiation and proliferation. We previously described that the transcription factor RIP140 was an important player in the regulation of intestinal homeostasis and tumorigenesis through the control of *APC* gene expression and activation of the Wnt pathway [27]. In the present study, we demonstrated that RIP140 also finely regulates the Notch signaling pathway and strongly cross‐talks with the HES1 transcription factor to regulate its own expression in a complex negative feedback regulatory loop (Fig. 9). This regulation of HES1 activity/expression has an impact on colon cancer cell proliferation and CRC prognosis.

By using different engineered human colorectal cell lines, we first identified RIP140 as a new modulator of RBPJ activity and, consequently, as a novel actor in the regulation of *HES1* gene expression in the intestinal epithelium (Fig. 9). RBPJ mediates gene transcription activation or repression depending on the protein complexes that are recruited. In the absence of nuclear NICD, RBPJ represses Notch target genes through the recruitment of corepressor complexes containing proteins such as HDACs, SMRT, NCoR, and/or CtBPs [8]. The positive regulation by RIP140 which takes place at the transcriptional level and involves the RBPJ binding site, might, at least partly, implicate squelching of HDACs or CtBPs since we previously reported that RIP140 directly interacted with these transcriptional repressors [20]. Concerning the induction of *HES1* gene expression by RIP140, we clearly showed that it implicates the RBPJ binding site present in the proximal region of the *HES1* promoter. However, we cannot exclude that RIP140 might activate *HES1* gene transcription through other signaling pathways such as the Wnt, Hedgehog, TGFβ/BMP or hypoxia pathways which can all affect Notch regulation of *HES1* gene expression [48].

Interestingly, RIP140 inhibits *HES1* gene transcription when the HES1 protein is highly expressed (see Fig. 9). In many types of cultured cells including fibroblasts, myoblasts, and neuroblasts, *HES1* exerts a negative feedback loop on its one expression leading to an autonomous oscillatory regulation of its expression with a periodicity of about 2 h. Hes1 thus acts as a biological clock capable of controlling the activation time of various biological processes such as cell cycle or differentiation [49, 50]. Interestingly, in pluripotent stem cells derived from the intern cellular mass of a blastocyst, HES1 oscillatory expression contribute to the multiple differentiation properties of these cells [13].

Our data indicate that RIP140 mainly acts through the amplification of the repressive activity that HES1 exert on its own promoter and, as a consequence, participates in the negative feedback loop that HES1 exerts on its own expression (Fig. 9). This effect might be a general one since it was also observed on the *HES4* gene whose expression is also repressed by HES1. Our data obtained using *in vitro* interaction, proximity ligation assays and co‐localization identify RIP140 as a new partner of HES1 and further work is needed to fully decipher the underlying mechanisms. It should be noted that little is known about the way HES1 represses gene expression. The C‐terminal WRPW domain of the protein interacts with TLE/Grg corepressors [51]. On the other hand, the bHLH domain of HES1 recruits Sirt1, a class III histone deacetylase, and thereby represses target gene expression [52].

The molecular machinery involved in the oscillation of *HES1* gene expression is not yet fully understood. It has been reported that the Jak–Stat signaling pathway [53] and the mir‐9 [54, 55] could be involved in this regulation. Yet, another mechanism used by HES1 to repress the expression of its target genes is by preventing the activity of transcription activators [49]. RIP140 could therefore titrate transcription activators, like ß‐catenin, and thus prevent their activity on the *HES1* gene promoter. More importantly, RIP140 is clearly engaged in a negative regulatory loop due to the induction of its expression by HES1. Although the HES1 transcription factor has been identified as a repressor of gene expression, it is also able to activate transcription. Several target genes induced by HES1 in CRC cells have been identified including the oncogene *BMI1* [56], which promotes invasion and migration of colon cancer cells.

In link with the numerous cross‐talks that occurred between the two transcription factors at different levels, we demonstrated that, in colon cancer cells, RIP140 is able to reverse the mitogenic effects of HES1 which switched to a repressor of cell proliferation in the presence of ectopic expression of RIP140. Based on these results, it is tempting to speculate that RIP140 might regulate other oncogenic functions of HES1 that have been identified such as its role in metastatic potentiation [57, 58]. This hypothesis is sustained by our observation in two cohorts of human CRC biopsies, in which we observed that the high coexpression of RIP140 and HES1 correlates with a better patient overall survival.

## 5 Conclusion

This study demonstrates for the first time that, in CRC cells, the transcription coregulator RIP140 (1) strongly cross‐talks and directly interacts with the HES1 transcription factor, (2) is engaged in the autoregulation of its oscillatory expression, (3) reverses its mitogenic activity on cancer cell proliferation, and (4) is required to reveal its correlation with prolonged patient survival. This work has few limitations linked mainly to the fact that we did not fully decipher the mechanism of the NICD/RBPJ mediated positive effect of RIP140 on HES1 expression (direct interaction with RBPJ/NICD or indirect mechanisms?) and neither had we demonstrated the requirement of RIP140 for the tumorigenic effect of HES1 *in vivo* using transgenic mice models. Nonetheless, this work strongly reinforces the role that RIP140 plays in intestinal tumorigenesis by controlling the Notch/HES1 signaling, a major molecular pathway involved in colon cancer.

## Conflict of interest

The authors declare no conflict of interest.

## Author contributions

NS, MK, SJ, SB, CT, SP, and ML performed the experiments. EB and AM helped to develop the methodology and experimental design. RY participated in transgenic animal generation. FB, NP, and FB‐M performed immunohistochemistry. FB‐M and AC‐N analyzed the immunohistochemistry data. VC conceptualized the study. NS and VC analyzed and interpreted the data. NS, ML, and VC generated figures and wrote the manuscript. All authors read and approved the final version of the manuscript.

### Peer review

The peer review history for this article is available at https://www.webofscience.com/api/gateway/wos/peer‐review/10.1002/1878‐0261.13626.

## Supporting information


**Fig. S1.** The *RIP140* gene is a target of the Notch/HES1 pathway.
**Fig. S2.** Correlation between RIP140 and HES1 expression in primary colorectal tumors and in normal adjacent tissues.
**Fig. S3.** The *RIP140* gene is a target of the Notch/HES1 pathway.
**Fig. S4.** RIP140 is required for the HES1 feedback loop.
**Fig. S5.** HES1/RIP140 interplay on intestinal tumorigenesis, CRC cell proliferation and patient survival.
**Table S1.** Primer sequences.

## Data Availability

The clinical data from patients are provided in Table 1. Primers list is provided in Table S1 (in the Supplementary Information file). The *in silico* data that support some of the findings of this study are available in The Cancer Genome Atlas (TCGA) portal at [http://tcga‐data.nci.nih.gov/docs/publications/coadread_2012/] and in the Cancertool database (http://genomics.cicbiogune.es/CANCERTOOL). All data that support the findings of this study are available within the article, its Supplementary Information, or from the corresponding author upon reasonable request.
