# Supplementary material for: RIP140 regulates transcription factor HES1 oscillatory expression and mitogenic activity in colon cancer cells
Source: Mol Oncol. 2024 Mar 8;18(6):1510–30. doi: 10.1002/1878-0261.13626 (PMC11161732; doi:10.1002/1878-0261.13626)
Supplement: Supplementary file 1 — Fig. S1. The RIP140 gene is a target of the Notch/HES1 pathway. Fig. S2. Correlation between RIP140 and HES1 expression in primary colorectal tumors and in normal adjacent tissues. Fig. S3. The RIP140 gene is a target of the Notch/HES1 pathway. Fig. S4. RIP140 is required for the HES1 feedback loop. Fig. S5. HES1/RIP140 interplay on intestinal tumorigenesis, CRC cell proliferation and patient survival. Table S1. Primer sequences. [file MOL2-18-1510-s001.zip › Sfeir et al_suppl_Mol Oncol_legend_check clean.docx]

**LEGEND TO SUPPLEMENTARY Figures**

**Figure S1:** The RIP140 gene is a target of the Notch/HES1 pathway.

**(A)** *RIP140* mRNA level in HT29 cells transiently transfected with HES1 expression vector or treated with siRNA targeting the *HES1* mRNA. Results are expressed as fold change ± standard-deviation (S.D.) relatively to controls; n = 4 independent experiments. **(B)** Luciferase reporter assay performed on the *RIP140* gene promoter construct (left panel) or on a reporter construct encompassing the very proximal region of *RIP140* gene promoter (right panel) transiently co-transfected into HT29 cells with the NICD expression vector. Relative luciferase unit (RLU) was expressed as mean ± SD; n = 3 independent experiments. **(C)** Luciferase reporter assay performed on *RIP140* gene promoter constructs transiently co-transfected into HT29 cells with the HES1 or DBD-HES1 expression vectors. RLU was expressed as mean ± SD; n = 3 independent experiments. **(D)** Luciferase reporter assay performed on a reporter construct encompassing the murine form of RIP140 promoter transiently co-transfected into SW620 or HT29 cells with HES1 expression vector. RLU was expressed as mean ± SD; n = 3 independent experiments. For all panels: ** p < 0.01 and *** p < 0.001 (Mann–Whitney test).

**Figure S2:** Correlation between RIP140 and HES1 expression at the mRNA level in primary colorectal tumors **(A)** and in normal adjacent tissues **(B)** after reanalysis of the Colonomics dataset using the Cancertool database.

**Figure S3:** The *RIP140* gene is a target of the Notch/HES1 pathway**.**

**(A)** *HES1* mRNA level in HT29 cells transiently transfected with increasing doses of RIP140 expression vector in the presence or not of a high dose of NICD expression vector. Results are expressed as fold change ± standard-deviation (S.D.) relatively to the control n = 3 independent experiments. **(B)** Luciferase reporter assay performed on *HES1* gene promoter (0.47kb) construct transiently co-transfected into HT29 cells with increasing doses of RIP140 expression vector in the presence or not of the NICD expression vector. Relative luciferase unit (RLU) was expressed as mean ± SD; n = 3 independent experiments. **(C)** *HES4* mRNA level in HT29 cells under the same conditions as in panel **(A)**. Results are expressed as fold change ± S.D. relatively to control; n = 3 independent experiments. **(D)** Luciferase reporter assay performed on the *HES1* gene promoter (0.47kb) construct transiently co-transfected into HT29 cells with doses of RIP140 and/or NICD expression vectors. RLU was expressed as mean ± SD; n = 3 independent experiments. For all panels: *** p < 0.001 (Mann–Whitney test).

**Figure S4:** RIP140 is required for the HES1 feed-back loop.

**(A)** Total (left panel) and endogenous (right panel) *HES1* gene expression distinctly detected using primers specific to the HES1 coding sequence and the 3’UTR of HES1 sequence, respectively in SW620 cells transiently transfected with increasing doses of LV-HES1 expression vector. Results are expressed as fold change ± standard-deviation (S.D.) relatively to the control; n = 3 independent experiments. **(B)** RT-qPCR analysis of *RIP140* mRNA level in SW620 cells transiently transfected with RIP140 expression vector or with the control siRNA (C) or siRNA targeting RIP140 (si-RIP140). Results are expressed as fold change ± S.D. relatively to the control; n = 3 independent experiments. **(C)** DuoLink proximity ligation assay performed to visualize endogenous HES1 and RIP140 interaction in HT29 cells. Scale bar: 50µm. The figure is representative of 3 independent experiments. **(D)** Double immunofluorescence analysis (40x) of HES1 and RIP140 protein levels in HT29 cells. Scale bar: 50µm. The figure is representative of 3 independent experiments.

**Figure S5:** HES1/RIP140 interplay on intestinal tumorigenesis, CRC cell proliferation and patient survival.

**(A)** Kaplan-Meier analysis performed on HES1 IHC data. Patients were ranked according to HES1 staining immunoreactive score (IRS) in their tumors and divided into two groups exhibiting low and high expression, respectively (best cut-off threshold). **(B)** and **(C)** Kaplan-Meier analysis of the cumulative overall survival (OS) of patients with low or high HES1 gene expression was performed using the Kaplan–Meier plotter database on the groups exhibiting low (panel B) or high (panel C) RIP140 gene expression. A log-rank test was used for statistical analysis. **(D)** HT29 cell proliferation was measured by xCELLigence assay after transfection or not of a siRNA targeting RIP140. n = 3 independent experiments.

**Table S1: Primer sequences**

| **Gene** | **Forward Sequence** | **Reverse Sequence** |
| --- | --- | --- |
| **hRIP140** | AATGTGCACTTGAGCCATGATG | TCGGACACTGGTAAGGCAGG |
| **hHES1** | AAGAAAGATAGCTCGCGGCAT | CCAGCACACTTGGGTCTGT |
| **hNotch1** | GAATGGTCAATGCGAGTGGC | GGCCCTGGTAGCTCATCATC |
| **hHES4** | TGGACGCCCTCAGAAAAGAG | TTCACCTCCGCCAGACACT |
| **hHES15’** | GGTGCTGATAACAGCGGAAT | TTGGAGTTCTTCACGAAAAAGA |
| **hp21** | TGAGCGATGGAACTTCGAC | ACAAGACAGTGACAGGTCC |
| **hp27** | AGCCTGGAGCGGATGGACGCC | CTCCCGCTGACATCCTGGCTC |
| **hMuc2** | GACACCATCTACCTCACCCG | TGTAGGCATCGCTCTTCTCA |
| **mRip140** | AGAACGCACATCAGGTGGCA | GATGGCCAGACACCCCTTTG |
| **mHES1** | GCGAAGGGCAAGAATAAATG | TGTCTGCCTTCTCTAGCTTGG |
| **mp21** | GTTCCGCACAGGAGCAAAGT | ACGGCGCAACTGCTCAC |
| **mMuc2** | CGACACCAGGGATTTCGCTTAAT | CACTTCCACCCTCCCGGCAAAC |
| **ComR1** | CCAGTGCTAGCATTCGCTGT | CTAAATCAGAACCCACCCCGGAT |
| **VillinCre** | CAAGCCTGGCTCGACGGCC | CGCGAACATCTTCAGGTTCT |
| **28S** | CGATCCATCATCCGCAATG | AGCCAAGCTCAGCGCAAC |
| **RS9** | CGGCCCGGGAGCTGTTGACG | CTGCTTGCGGACCCTAATGTGACG |
| **ChIP HES1 prom** | GCGTGTCTCCTCCTCCCATT | CCTGGCGGCCTCTATATATA |
