# Supplementary figures and images for: RIP140 regulates transcription factor HES1 oscillatory expression and mitogenic activity in colon cancer cells
Source: Mol Oncol. 2024 Mar 8;18(6):1510–30. doi: 10.1002/1878-0261.13626 (PMC11161732; doi:10.1002/1878-0261.13626)

**A**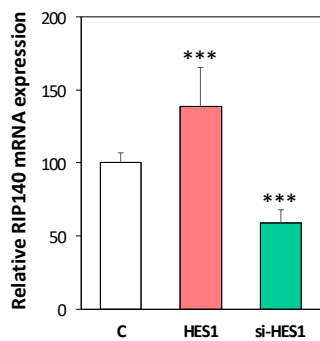**B**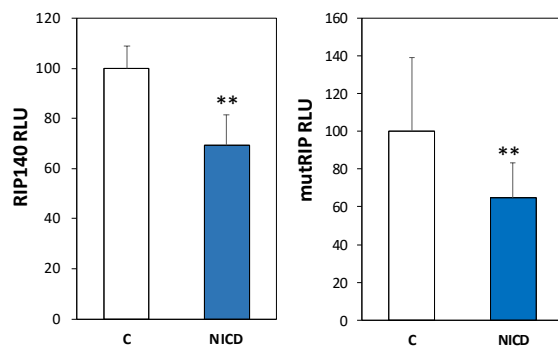**C**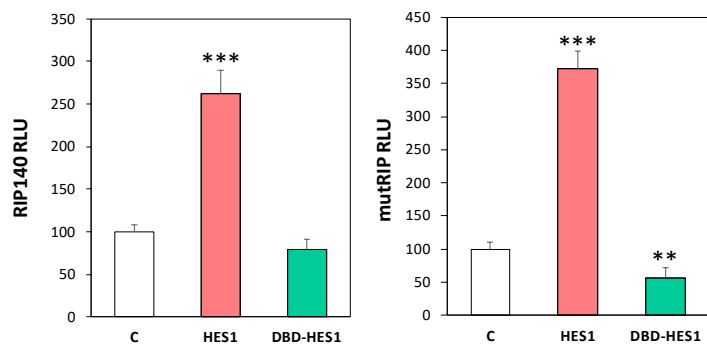**D**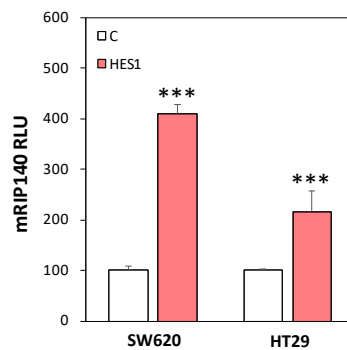**Figure S1**

**A**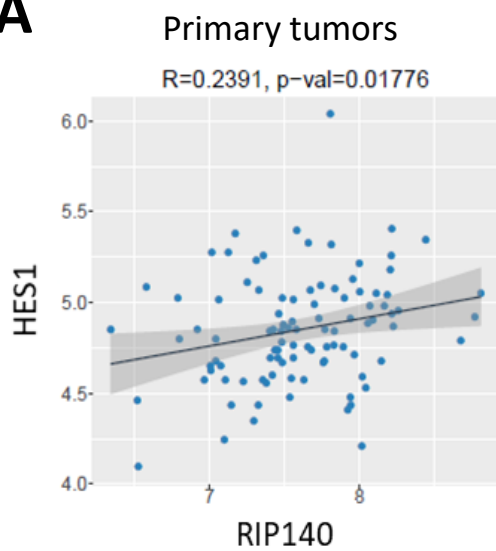**B**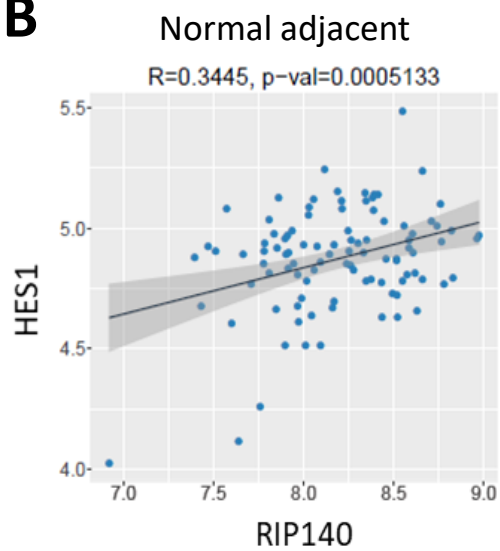**Figure S2**

**A**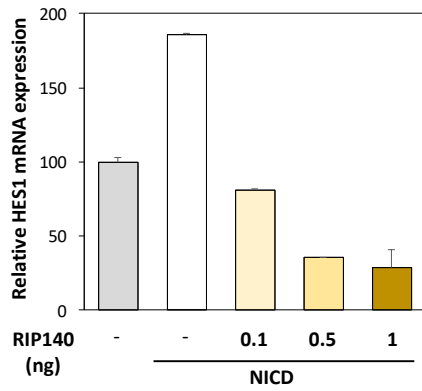**B**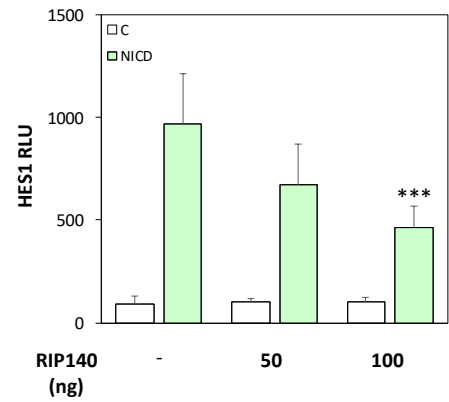**C**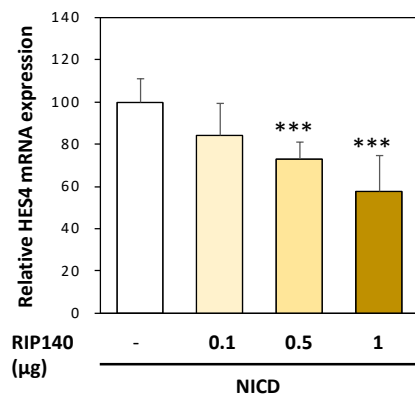**D**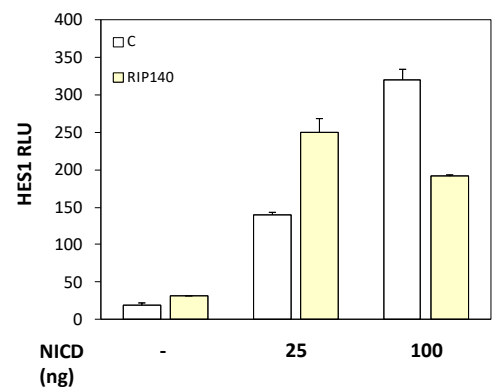**Figure S3**

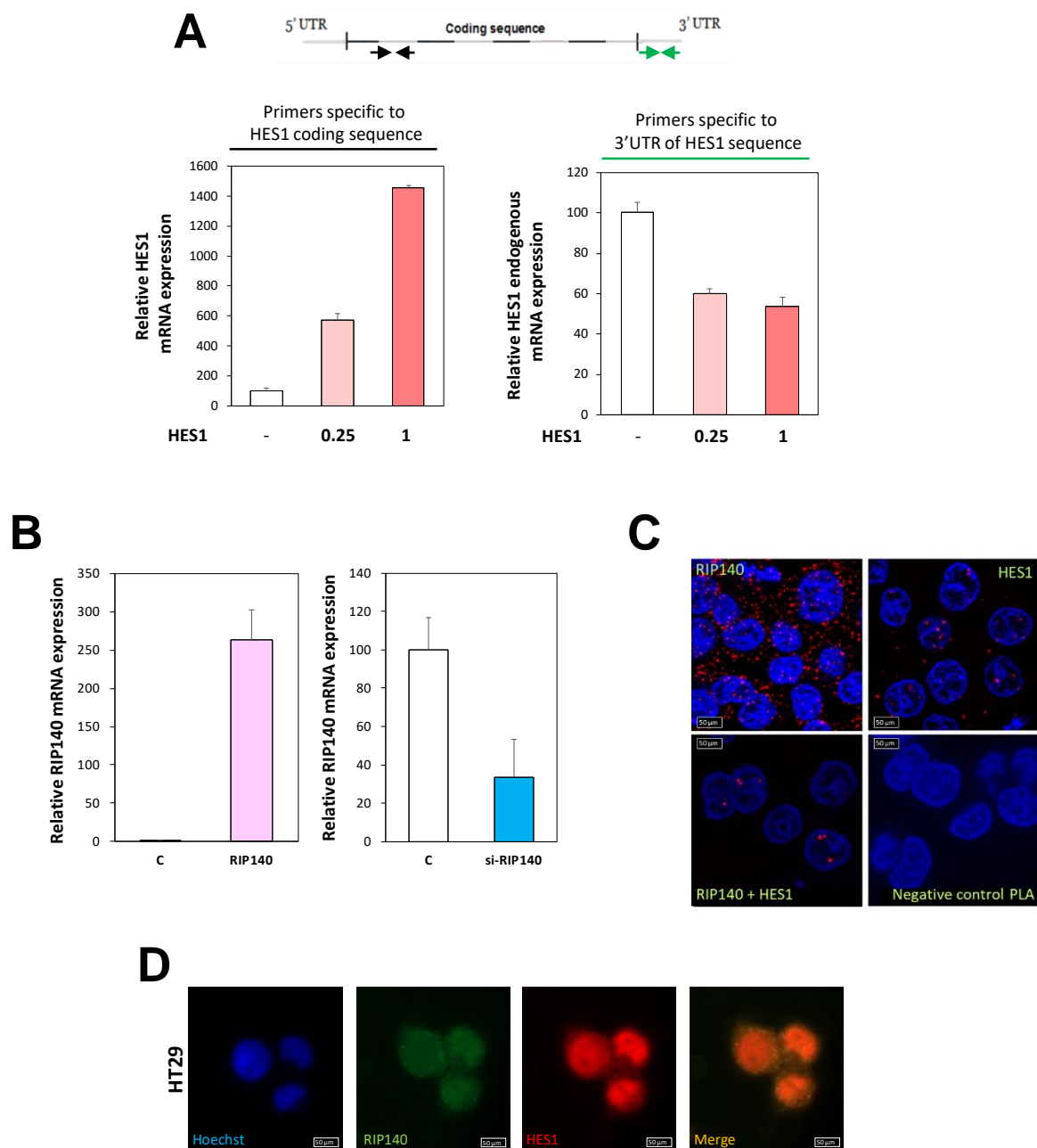

Figure S4

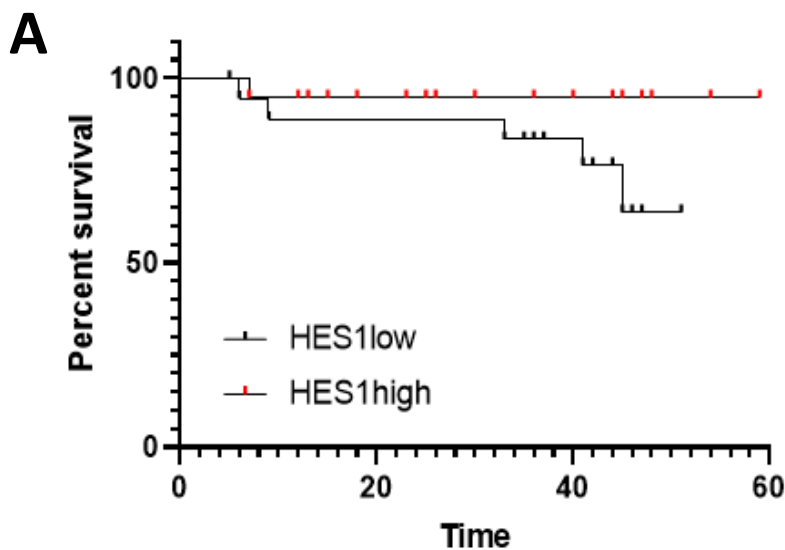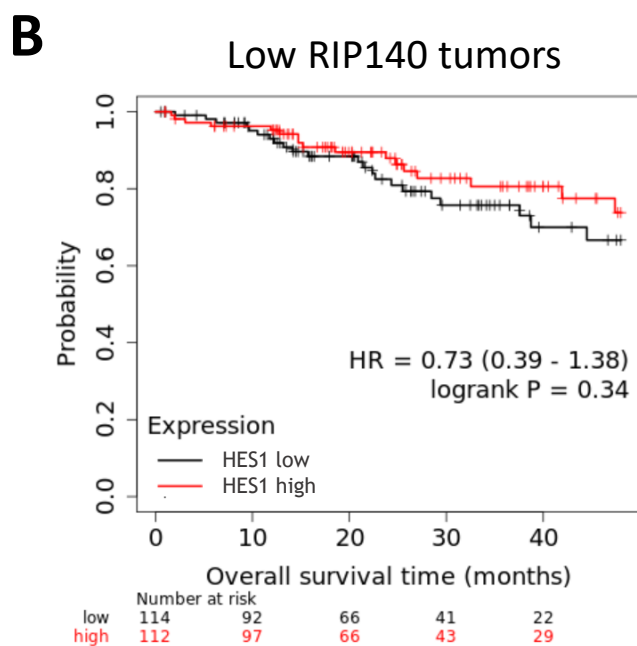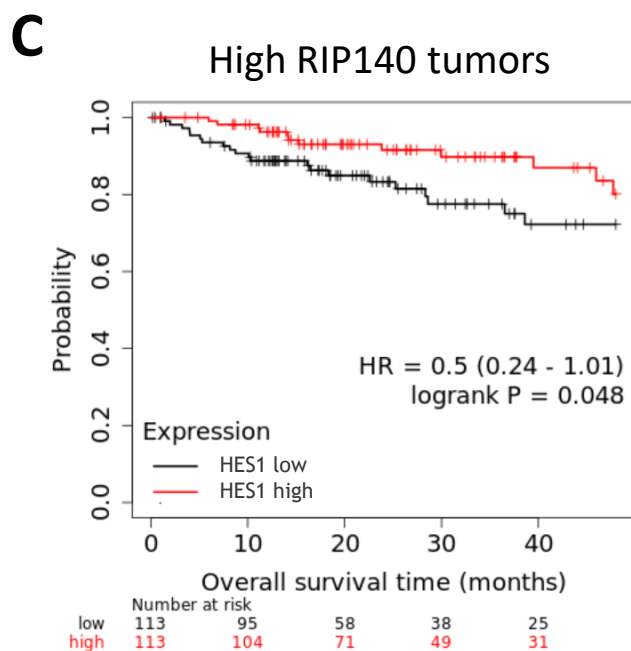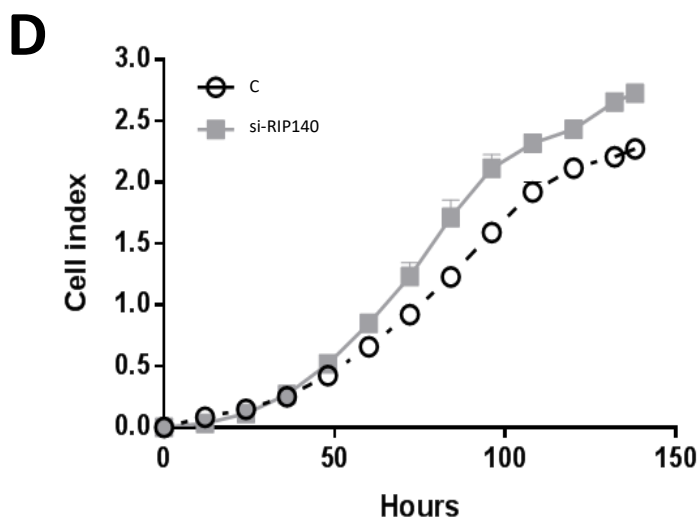

**Figure S5**

Supplement: Supplementary file 1 — Fig. S1. The RIP140 gene is a target of the Notch/HES1 pathway. Fig. S2. Correlation between RIP140 and HES1 expression in primary colorectal tumors and in normal adjacent tissues. Fig. S3. The RIP140 gene is a target of the Notch/HES1 pathway. Fig. S4. RIP140 is required for the HES1 feedback loop. Fig. S5. HES1/RIP140 interplay on intestinal tumorigenesis, CRC cell proliferation and patient survival. Table S1. Primer sequences. [file MOL2-18-1510-s001.zip › Figures Suppl Sfeir at al. accepted_check.pdf]
